# The *COL4A3* c.3418+1G>T variant causes exon 39 skipping in minigene assays: functional and exploratory structural analyses

**DOI:** 10.3389/fgene.2026.1953498

**Published:** 2026-10-13

**Authors:** Xuan Ban, Yue Wang, Yunshan Guo, Juan Wang, He Sun, Ping He, Mingxiu Wang, Xiaofang Tang, Qiuling Fan

**Affiliations:** 1 Department of Nephrology, Shanghai General Hospital, Shanghai Jiao Tong University School of Medicine, Shanghai, China; 2 Department of Endocrinology, Shengjing Hospital of China Medical University, Shenyang, China; 3 Precision Research Center for Refractory Diseases, Shanghai General Hospital, Shanghai Jiao Tong University School of Medicine, Shanghai, China

**Keywords:** ACMG variant classification, alport syndrome, autosomal dominant alport syndrome, *COL4A3*, exon skipping, minigene assay, splice-site variant, type IV collagen

## Abstract

**Background:**

Canonical splice-site variants are an important cause of Alport syndrome, but the transcript consequences of many heterozygous *COL4A3* variants remain experimentally unverified. We characterized *COL4A3* c.3418 + 1G>T in a 38-year-old woman with persistent isolated microscopic hematuria. Notably, the proband’s mother also had microscopic hematuria but did not carry c.3418 + 1G>T, precluding supportive co-segregation.

**Methods:**

Whole-exome sequencing identified the variant, which was subsequently confirmed by Sanger sequencing. Splicing effects were assessed using SpliceAI, Pangolin, and MaxEntScan. Minigene assays were performed in HEK293T, HK-2, and human glomerular endothelial cells. Protein consequences were examined using AlphaFold-derived models, PyMOL visualization, and exploratory 5-ns molecular dynamics simulations of local α3α4α5(IV) trimer models, with additional independently initialized 20-ns replicate simulations performed as a supplementary reproducibility analysis.

**Results:**

Computational tools consistently predicted loss of the canonical donor splice site. Minigene assays demonstrated complete exon 39 skipping in all 3 cell lines, resulting in an 81-bp in-frame deletion of 27 amino acids within the α3(IV) collagenous domain. Sequence examination showed that the resulting junction preserved the Gly-X-Y register, although its protein-level structural consequences remain undetermined. Exploratory molecular dynamics analysis showed descriptive differences between wild-type and mutant models. The additional 20-ns simulations showed broadly similar RMSD ranges across independent replicates, although RMSD values varied across prespecified time blocks, indicating limited short-timescale sampling consistency. These findings were therefore considered hypothesis-generating rather than evidence of full convergence, altered stability, or impaired interchain interactions. The patient exhibited persistent isolated microscopic hematuria without proteinuria, renal dysfunction, or extrarenal manifestations, compatible with a mild autosomal dominant Alport syndrome phenotype.

**Conclusion:**

*COL4A3* c.3418 + 1G>T causes complete exon 39 skipping in the tested minigene systems and a 27-amino-acid in-frame deletion. Under a conservative author-level application of the ACMG/AMP framework, PVS1_Moderate and PM2_Supporting yield a primary classification of Variant of Uncertain Significance (VUS); PP4 was not applied in the primary classification because isolated microscopic hematuria has limited specificity and the maternal phenotype was discordant. A Likely Pathogenic classification is retained only as a conditional author-level alternative if PVS1 (RNA) is calibrated to Strong and PP4 is applied.

## 1 Introduction

Alport syndrome (AS) is an inherited disorder of the glomerular basement membrane (GBM) caused by pathogenic variants in *COL4A3*, *COL4A4*, or *COL4A5*, which encode the α3, α4, and α5 chains of type IV collagen, respectively (Kashtan et al., 2018; Savige et al., 2022). These three chains assemble into the α3α4α5 collagen IV heterotrimer, an essential structural component responsible for maintaining GBM integrity and filtration function (Kashtan et al., 2018; Naylor et al., 2021; Savige et al., 2022). Disruption of this collagen network leads to persistent hematuria, progressive proteinuria, chronic kidney disease, sensorineural hearing loss, and ocular abnormalities. Depending on the underlying genetic defect, AS may be inherited in X-linked, autosomal recessive, or autosomal dominant forms. Recent advances in molecular genetics have shown that some individuals previously diagnosed with thin basement membrane nephropathy actually represent the mild end of the autosomal dominant Alport syndrome spectrum (Kashtan et al., 2018; Savige et al., 2022).

Splice-site variants constitute an important class of pathogenic variants in collagen IV genes and frequently result in exon skipping, intron retention, or activation of cryptic splice sites (Chen et al., 2025; Daga et al., 2025). Among these mechanisms, canonical splice-site variants affecting the conserved ± 1 or ± 2 nucleotides are generally predicted to abolish normal splicing and may therefore provide important pathogenic evidence under current variant interpretation guidelines (Richards et al., 2015). However, because computational prediction alone cannot determine whether a variant causes exon skipping, partial exon deletion, cryptic splice-site activation, or another alternative splicing event, experimental transcript-level validation remains important for defining its actual RNA consequence and informing variant classification (Chen et al., 2025; Daga et al., 2025).

The *COL4A3* c.3418 + 1G>T variant is located at the highly conserved donor splice site immediately downstream of exon 39. This variant was previously reported in a Chinese family and co-segregated in cis with *COL4A4* c.1471C>T in a benign familial hematuria phenotype (Li et al., 2018). However, its transcript-level consequence has not been experimentally characterized, and its molecular mechanism as an apparently isolated heterozygous finding remains unclear (Li et al., 2018). Moreover, the structural implications of exon 39 skipping on collagen IV assembly have not been directly investigated.

In the present study, we combined computational splice prediction, minigene assays performed in 3 cell lines, and AlphaFold-based structural interpretation to investigate the molecular consequence of *COL4A3* c.3418 + 1G>T. We further explored the potential structural implications of exon 39 skipping and assessed the variant using current ACMG/AMP, Alport-specific, and ClinGen SVI splicing guidance.

## 2 Materials and methods

### 2.1 Patient and clinical evaluation

Clinical information was retrospectively obtained from medical records, laboratory results, imaging examinations, family history, and follow-up records. Clinical evaluation included urinalysis with urinary erythrocyte morphology, urinary protein and albumin excretion, serum creatinine, blood pressure, computed tomography urography, and hearing and ocular assessments. Available family members were evaluated using clinical history and targeted genetic testing when samples were available. The study was approved by the Institutional Ethics Committee of Shanghai General Hospital (approval no. 2026KS502) and was conducted in accordance with the Declaration of Helsinki. Written informed consent for participation and publication was obtained from the proband and her mother.

### 2.2 Whole-exome sequencing and variant confirmation

Genomic DNA was extracted from peripheral blood using standard procedures. Exonic regions and flanking splice sites were captured using the Roche KAPA HyperExome kit, followed by high-throughput sequencing on an MGI platform. Sequencing reads were aligned to the GRCh37/hg19 reference genome using BWA, and single-nucleotide variants and small insertions/deletions were called using GATK. Variants were prioritized according to population frequency, predicted functional consequence, inheritance pattern, and phenotypic concordance. Candidate variants were confirmed by Sanger sequencing in the proband, and segregation analysis was performed in available family members.

### 2.3 In silico splicing analysis

Potential effects on pre-mRNA splicing were evaluated using three independent algorithms: SpliceAI, Pangolin, and MaxEntScan. Prediction results were interpreted mainly according to disruption of the canonical donor splice site and the likelihood of cryptic splice-site activation.

### 2.4 Minigene construction and splicing assay

To experimentally evaluate RNA splicing, genomic fragments containing *COL4A3* exon 39 and flanking intronic sequences were amplified and cloned into the pSPL3 exon-trapping vector. Wild-type and mutant constructs carrying the c.3418 + 1G>T substitution were generated by site-directed mutagenesis.

The constructs were transiently transfected into HEK293 T cells, HK-2 renal tubular epithelial cells, and human glomerular endothelial cells (HGECs). Total RNA was isolated 24 h after transfection and reverse transcribed into cDNA. Conventional RT-PCR was performed using vector-specific primers and 2× Taq Master Mix (Dye Plus) (Vazyme, Nanjing, China; P112), with an initial denaturation at 95 °C for 5 min, followed by 32 cycles of 95 °C for 15 s, 50 °C for 15 s, and 72 °C for 60 s/kb, and a final extension at 72 °C for 5 min. PCR products were analyzed by agarose gel electrophoresis and confirmed by Sanger sequencing. To assess the steady-state abundance of minigene-derived transcripts, RT-qPCR was additionally performed using primers targeting a pSPL3-derived exonic region shared by the WT and MUT transcripts. Relative transcript abundance was calculated using the 2^−ΔΔCt^ method with ACTB as the reference gene and a WT sample as the calibrator. Two independent transfection experiments were analyzed for each cell line. Primer sequences used for minigene RT-PCR, minigene-derived transcript RT-qPCR, and sequencing are provided in Supplementary Table S1.

### 2.5 Structural interpretation

The transcript consequence identified by the minigene assay was translated *in silico* to generate the corresponding mutant α3(IV) amino-acid sequence for structural analysis. The predicted full-length wild-type α3(IV) chain model (UniProt Q01955) was obtained from the AlphaFold Protein Structure Database. The corresponding mutant α3(IV) sequence was submitted to AlphaFold for structural prediction. The wild-type and mutant α3(IV) monomer models were subsequently visualized and compared in PyMOL to evaluate the localization of the deleted region within the α3(IV) collagenous domain and its effect on local junction formation. For visualization in a three-chain context and subsequent exploratory molecular dynamics analysis, WT and MUT α3α4α5(IV) local trimer models were generated using AlphaFold Server.

### 2.6 Exploratory molecular dynamics analysis

To explore the potential conformational consequences of the minigene-defined sequence alteration, short-term exploratory molecular dynamics simulations were performed using GROMACS 2025.4 on AlphaFold Server-derived WT and MUT local α3α4α5(IV) trimer models centered on the affected region. The CHARMM36 m protein parameters, as implemented in the CHARMM36 July 2022 GROMACS distribution, and the accompanying CHARMM TIP3P water model were used. Each system was solvated in a rectangular box, neutralized, and adjusted to approximately 0.15 M NaCl. Following energy minimization and NVT/NPT equilibration at 310 K and 1 bar, the descriptive analysis presented in Supplementary Figure S7 was based on one 5-ns trajectory for each model, using a 2-fs time step under periodic boundary conditions. After PBC correction, protein centering, and backbone fitting, the trajectories were analyzed using backbone RMSD, radius of gyration (Rg), chain-specific Cα RMSF, and raw local inter-chain heavy-atom contact counts within 5 Å. Because the WT and MUT contact selections differed in residue and atom composition, the contact counts were not normalized and were interpreted descriptively rather than as quantitative measures of interaction strength. To further assess the reproducibility of these exploratory observations, three independently initialized 20-ns trajectories were additionally generated for each WT and MUT model. For the replicate analyses, trajectories were reconstructed to maintain the three-chain trimer across periodic boundaries before centering and fitting. Sequence-matched common-core backbone RMSD trajectories, matched α3(IV) Cα RMSF profiles, and prespecified RMSD summaries over the 5–10, 10–15, and 15–20 ns intervals were then examined as an extended short-timescale reproducibility analysis. These additional analyses are presented in Supplementary Figures S8–S9 and Supplementary Table S3 and were not used to replace or quantitatively validate the descriptive 5-ns analysis shown in Supplementary Figure S7. Detailed model construction, simulation parameters, and analysis selections are provided in Supplementary Method S1.

### 2.7 Variant classification

Variant classification was performed using the ACMG/AMP sequence variant interpretation framework together with the Alport syndrome-specific refinements for *COL4A3*–*COL4A5* (Savige et al., 2021). Evidence available from the initial whole-exome sequencing report was compared with the transcript-level evidence generated in the present study. Following the 2023 ClinGen SVI splicing recommendations, experimentally observed RNA-splicing consequences may be incorporated using PVS1(RNA), with the evidence strength calibrated according to the predicted protein consequence under the PVS1 decision framework; PS3 should not be applied separately to the same RNA result because this would double-count a single line of evidence (Walker et al., 2023). For an in-frame splicing outcome, Strong strength may be applied when the altered region is considered critical to protein function, whereas Moderate strength is generally appropriate when the functional importance of the affected region is uncertain and less than 10% of the protein is removed (Abou Tayoun et al., 2018). Evidence combinations before and after the minigene assay were compared using primary and conditional classification frameworks based on PVS1(RNA) strength and PP4 application. This was an author-level assessment and was not intended to replace an accredited clinical laboratory report.

## 3 Results

### 3.1 Clinical presentation

The proband was a 38-year-old woman with a 10-year history of persistent isolated microscopic hematuria. Urinary erythrocyte morphology demonstrated predominantly dysmorphic erythrocytes, indicating glomerular bleeding. Computed tomography urography showed no structural abnormalities of the urinary tract. No proteinuria, hypertension, renal insufficiency, hearing impairment, or ocular abnormalities were observed. Kidney function remained normal throughout follow-up. Relevant urinalysis and serum renal function findings are summarized in Tables 1, 2. The proband’s mother had a long-standing history of microscopic hematuria but did not carry the *COL4A3* c.3418 + 1G>T variant. The proband’s father was reported by family members to have no known renal manifestations; however, contemporaneous urinalysis, serum creatinine measurements, other formal renal evaluation results, and a paternal DNA sample were unavailable (Figure 1A). Therefore, normal renal status could not be confirmed. Consequently, the available family data did not establish co-segregation or a *de novo* occurrence. Accordingly, PP1 and PS2/PM6 were not applied in the variant classification. The proband’s clinical findings were compatible with a mild phenotype within the autosomal dominant *COL4A3*-related disease spectrum.

**TABLE 1:** Urinalysis findings in the proband.

Characteristic	Result	References range
Urine albumin-to-creatinine ratio (UACR)	8.90	<30 μg/mg
Total 24-h urinary protein	0.14	<0.15 g/24 h
Urinary microalbumin	17.53	<30 mg/L
Urinary erythrocytes	402↑	0–17/μL

The upward arrow (↑) indicates a value above the reference range.

**TABLE 2:** Serum renal function findings in the proband.

Characteristic	Result	References range
Urea	5.20	2.5–7.1 mmol/L
Creatinine	47.7	46–92 μmol/L

**FIGURE 1:**
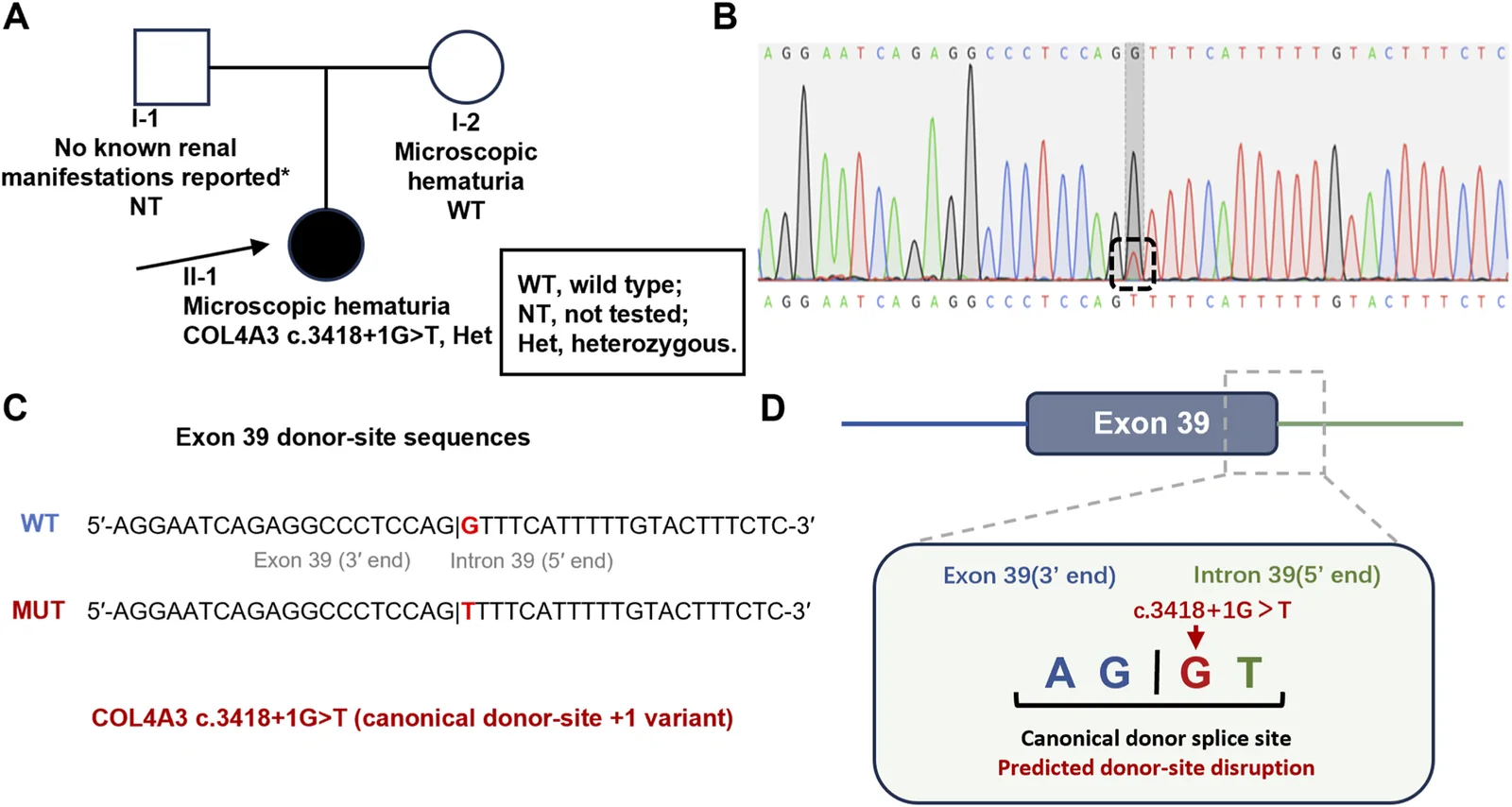
Clinical pedigree, Sanger validation, and splice-site localization of the *COL4A3* c.3418 + 1G>T variant. **(A)** Pedigree and available clinical/genetic information. II-1 is the proband. The proband’s father (I-1) was reported by the family to have no known renal manifestations; the asterisk indicates that contemporaneous urinalysis and renal function assessment were unavailable, and NT indicates that genetic testing was not performed. The proband’s mother (I-2) had a history of microscopic hematuria but did not carry c.3418 + 1G>T (WT). Thus, the available family data did not establish co-segregation or a *de novo* occurrence. **(B)** Sanger sequencing of the proband showing the heterozygous *COL4A3* c.3418 + 1G>T substitution. The dashed box indicates the variant position. **(C)** Single-stranded 5′-to-3′ sequence comparison of the wild-type (WT) and mutant (MUT) alleles at the exon 39-intron 39 junction. The vertical bar indicates the exon–intron boundary, and the highlighted nucleotides denote the invariant +1 G in WT and the substituted T in MUT. **(D)** Schematic representation of the canonical donor splice site at the exon 39-intron 39 junction. The variant affects the +1 G of the AG|GT donor motif and is predicted to disrupt donor-site recognition. WT, wild type; MUT, mutant; NT, not tested; Het, heterozygous.

### 3.2 Identification of a canonical *COL4A3* splice-site variant

Whole-exome sequencing identified a heterozygous canonical splice-site variant, *COL4A3* c.3418 + 1G>T (NM_000091.5), which alters the invariant +1 guanine of the canonical GT dinucleotide at the 5′donor splice site immediately downstream of exon 39. Review of the proband’s WES data showed that *COL4A4* c.1471C>T, previously reported in cis with *COL4A3* c.3418 + 1G>T by Li et al. (2018), was not detected. Sanger sequencing confirmed the heterozygous substitution (Figure 1B). The corresponding Sanger traces for the proband and her mother are provided in Supplementary Figure S1. Local WT/MUT sequence alignment and splice-site schematic further showed that the variant was located at the canonical 5′donor splice site at the exon 39-intron 39 junction and directly altered the +1 G of the AG|GT donor motif (Figures 1C,D). The variant is located within the collagenous domain-encoding region of *COL4A3* and therefore has the potential to alter the structure of the α3(IV) collagen chain.

### 3.3 Computational prediction indicates disruption of the canonical donor splice site

All three prediction algorithms consistently suggested severe disruption of the native donor splice site (Figure 2). SpliceAI predicted a strong donor-loss signal (Δscore = 0.85) and an acceptor-loss signal (Δscore = 0.63) (Figure 2A), whereas Pangolin similarly indicated marked splice-site loss (Δscore = 0.56) (Figure 2B). MaxEntScan analysis of the native 5′donor splice site showed a marked reduction in splice-site strength after the G>T substitution (ΔMaxEnt score [MUT−WT] = −8.5) (Figure 2C). In addition, the predicted splice pattern suggested that the variant may lead to exon 39 skipping and an 81-bp deletion (Figure 2D). Collectively, these computational analyses suggested that c.3418 + 1G>T abolishes normal donor splice-site recognition and is likely to induce abnormal RNA splicing. Detailed graphical outputs of the splice-prediction analyses are provided in Supplementary Figures S2 and S3.

**FIGURE 2:**
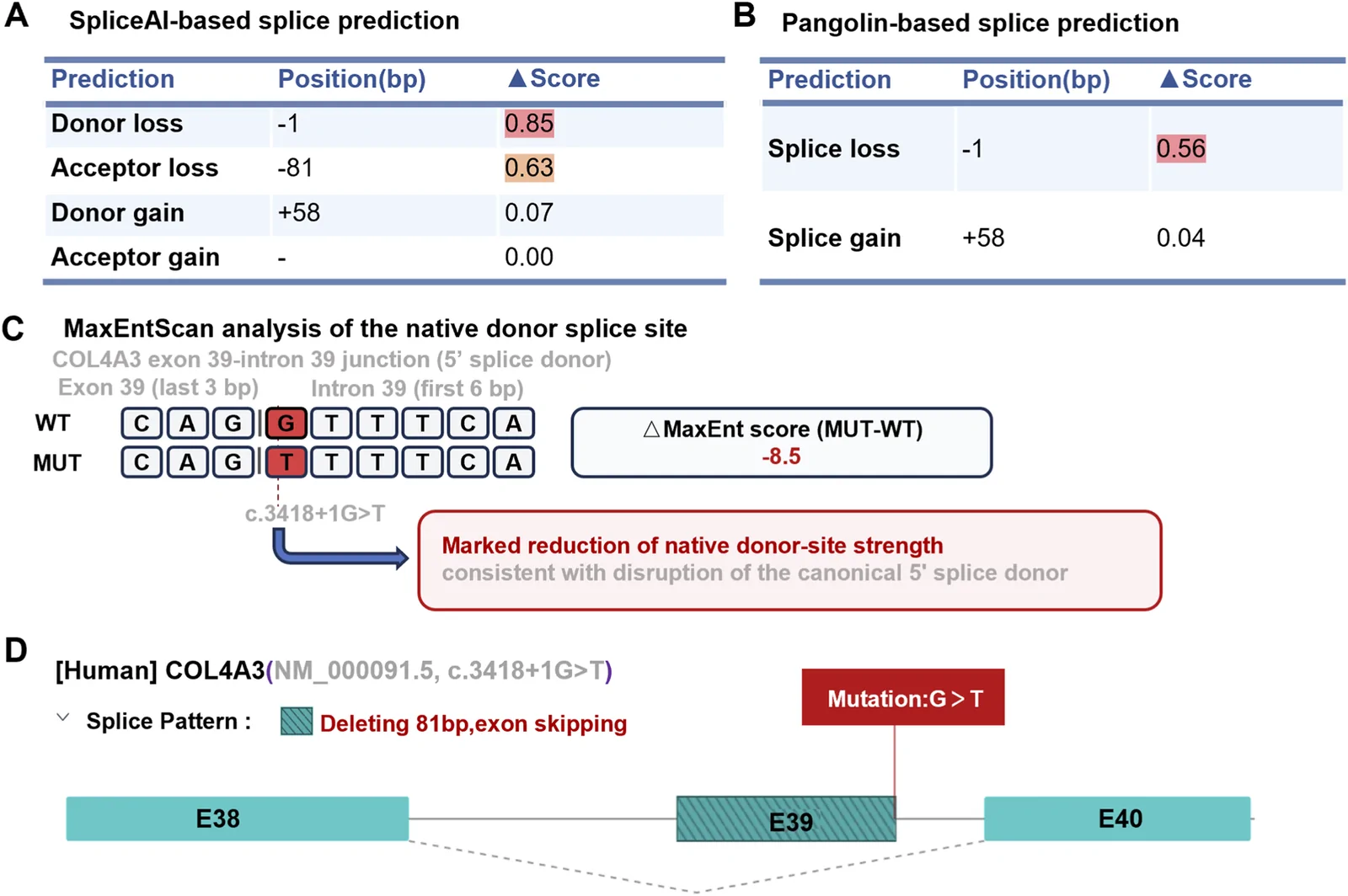
In silico prediction of the splicing effect of *COL4A3* c.3418 + 1G>T. **(A)** SpliceAI prediction showing a predominant donor-loss signal at position −1, together with an acceptor-loss signal at position −81. **(B)** Pangolin prediction showing splice loss at position −1. **(C)** MaxEntScan analysis of the native 5′splice donor at the exon 39–intron 39 junction, showing that the G>T substitution at the canonical +1 position markedly reduced donor-site strength (ΔMaxEnt score [MUT−WT] = −8.5). The two vertical lines indicate the exon 39–intron 39 boundary, and the canonical +1 nucleotide is highlighted in the wild-type and mutant sequences. **(D)** Predicted splice pattern indicating exon 39 skipping, resulting in an 81-bp deletion. Together, these computational predictions consistently support disruption of the native 5′splice donor site by the c.3418 + 1G>T variant. WT, wild type; MUT, mutant.

### 3.4 Functional validation demonstrates complete exon 39 skipping

Minigene assays were subsequently performed to experimentally determine the transcript consequence of the variant. WT/MUT fragments containing *COL4A3* exon 39 and flanking intronic sequences were cloned into the pSPL3 vector and transfected into HEK293T, HK-2, and HGECs, followed by RNA extraction, reverse transcription, RT-PCR, gel electrophoresis, and Sanger sequencing (Figure 3A).

**FIGURE 3:**
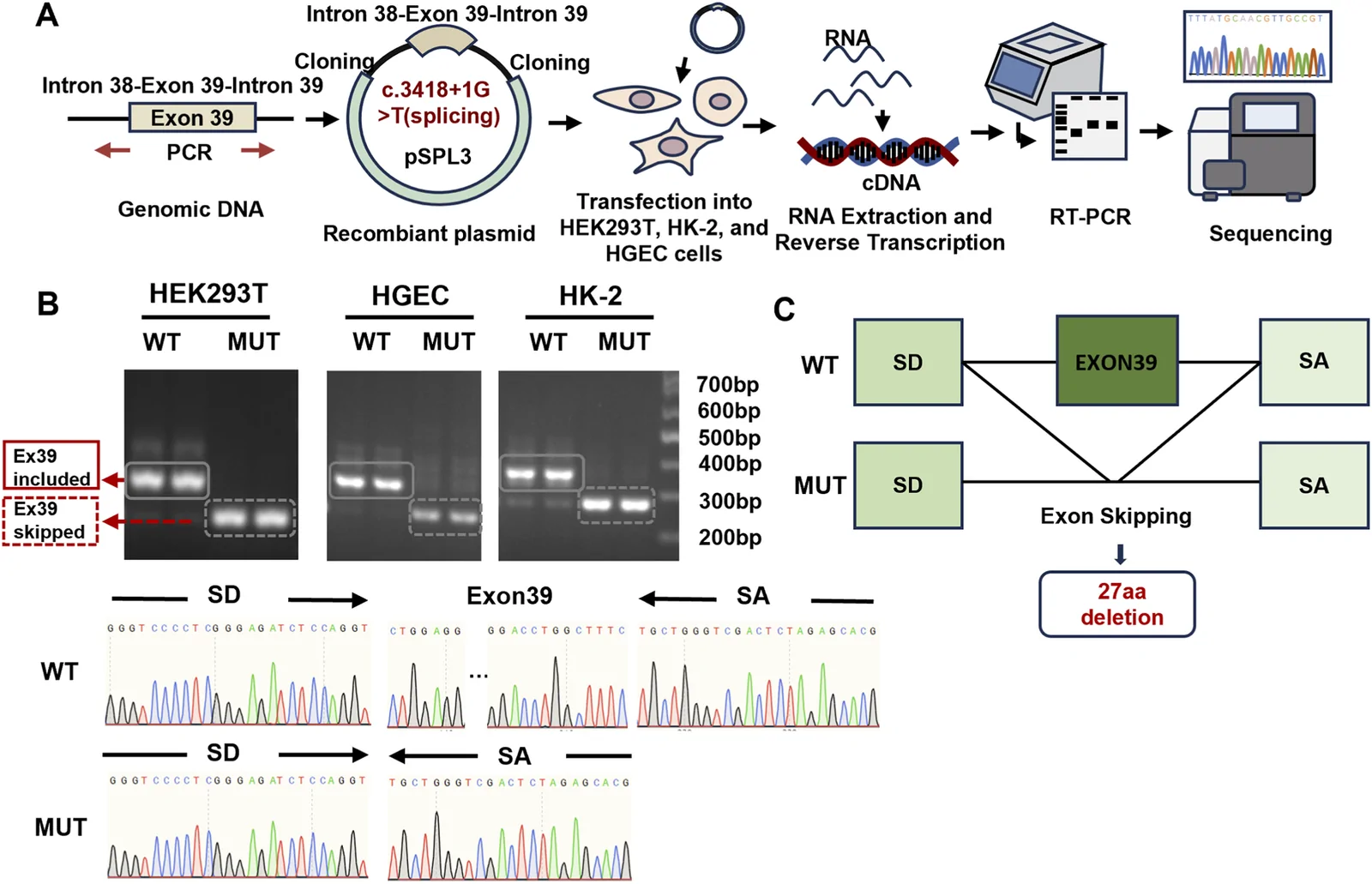
Minigene assay confirming exon 39 skipping caused by *COL4A3* c.3418 + 1G>T. **(A)** Workflow of the minigene assay. The genomic fragment containing *COL4A3* exon 39 and flanking intronic sequences was cloned into the pSPL3 vector, transfected into HEK293T, HK-2, and HGECs, and analyzed by RT-PCR and Sanger sequencing. **(B)** Agarose gel electrophoresis and Sanger sequencing of RT-PCR products. In all 3 cell lines, the upper bands correspond to the exon 39-included WT minigene products, whereas the lower bands correspond to the exon 39-skipped MUT products (Δ81 bp). Sanger sequencing confirmed complete exon 39 skipping in the mutant constructs. **(C)** Schematic representation of the splicing pattern in the pSPL3 system. The c.3418 + 1G>T mutant construct causes exon 39 skipping, leading to an 81-bp in-frame deletion corresponding to a predicted 27-amino-acid deletion in the encoded protein. WT, wild type; MUT, mutant; SD, splice donor; SA, splice acceptor; RT-PCR, reverse-transcription polymerase chain reaction; HGECs, human glomerular endothelial cells.

Wild-type constructs generated the expected transcript containing exon 39, whereas mutant constructs consistently produced a shorter transcript in HEK293T, HK-2, and HGECs (Figure 3B). Sanger sequencing confirmed complete skipping of exon 39 in all 3 cell lines. Identical splicing outcomes were observed in all three tested cell lines, supporting the robustness of exon 39 skipping across the tested cellular contexts. Additional results from HGECs and HK-2 cells are provided in Supplementary Figure S4, and the full original agarose gel image, including all lanes and molecular-weight markers, is provided in Supplementary Figure S5.

The aberrant transcript resulted in an 81-bp in-frame deletion corresponding to 27 amino acids while preserving the translational reading frame (Figure 3C). An exploratory RT-qPCR assessment showed some variation in minigene-derived transcript abundance between WT and MUT constructs, but no consistent marked depletion of the MUT transcript was observed across the 3 cell lines (Supplementary Figure S6).

### 3.5 Structural interpretation of the exon 39 skipping-induced in-frame deletion

To investigate the structural consequence of exon 39 skipping, we generated wild-type and mutant α3(IV) amino-acid sequences based on the experimentally confirmed transcript result. The mutant sequence harbored the in-frame deletion p. Lys1114_Gly1140del (Δ27 aa). The wild-type and mutant α3(IV) models were subsequently visualized and compared in PyMOL (Figure 4B). Protein domain schematics showed that p. Lys1114_Gly1140del was located within the collagenous triple-helical domain of the α3(IV) chain and upstream of the NC1 domain (Figure 4A).

**FIGURE 4:**
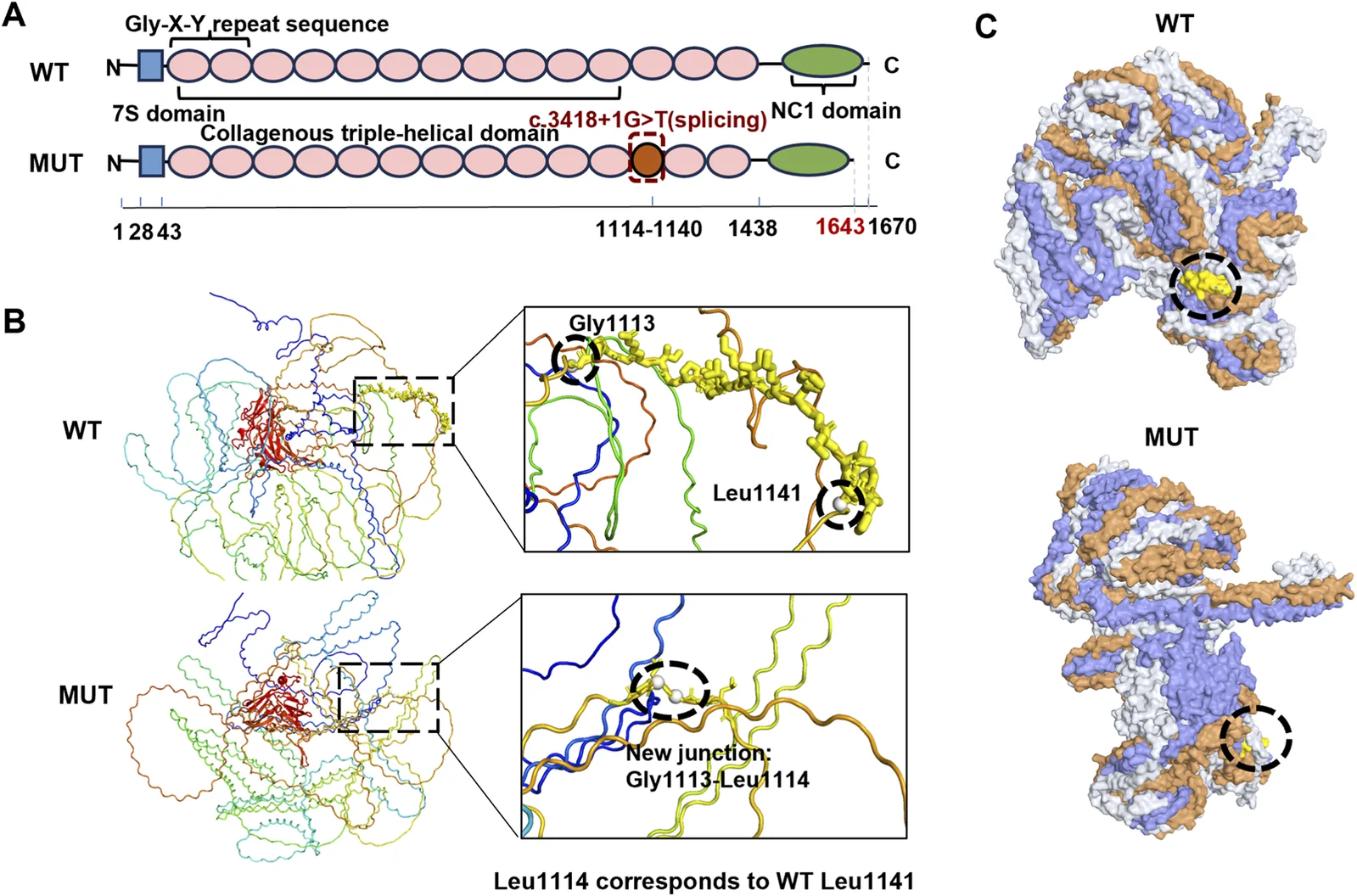
Structural visualization of the *COL4A3* c.3418 + 1G>T-associated exon 39 skipping event. **(A)** Schematic localization of the exon 39 skipping-induced in-frame deletion within the *COL4A3*-encoded α3(IV) chain. The deletion corresponds to p. Lys1114_Gly1140del (Δ27 aa) within the collagenous domain. **(B)** AlphaFold-predicted wild-type and mutant α3(IV) monomer models visualized in PyMOL. In the mutant model, wild-type Gly1113 is joined to wild-type Leu1141, which is renumbered as Leu1114 in the mutant protein. The resulting junctional sequence, Gly1113-Leu1114-Pro1115-Gly1,116, retains the Gly-X-Y register. **(C)** Visualization of the affected region in the α3α4α5 collagen IV heterotrimer context, showing the spatial location of the deletion-associated region within the three-chain assembly.

The deleted segment was 27 amino acids long, equivalent in length to nine tripeptide units. Sequence examination based on the reference α3(IV) protein sequence (UniProt Q01955) showed that deletion of Lys1114-Gly1,140 joined Gly1113 to wild-type Leu1141-Pro1142-Gly1,143. After renumbering of the mutant protein, the resulting sequence was Gly1113-Leu1114-Pro1115-Gly1,116, which retained a Gly-X-Y-Gly pattern and therefore did not shift the periodic glycine register. The trimer model localized the deletion-associated region within the spatial context of the α3α4α5(IV) heterotrimer (Figure 4C). However, structural prediction and visualization alone cannot determine whether the 27-amino-acid shortening alters local interchain alignment, triple-helical packing, secretion, or heterotrimer assembly.

### 3.6 Exploratory molecular dynamics analysis shows descriptive differences between WT and MUT models

To further examine the possible dynamic consequences of the in-frame deletion in the trimer context, one 5-ns WT trajectory and one 5-ns MUT trajectory of the local trimer models were descriptively compared. After PBC correction, protein centering, and backbone fitting, the RMSD trajectories showed different conformational profiles relative to their respective starting reference frames (Supplementary Figure S7A). Because the two systems were derived from different starting models and no replicate-level statistical analysis was performed, these profiles were not interpreted as a quantitative comparison of structural stability. The Rg trajectories also differed; however, this difference may partly reflect the shorter α3(IV) chain in the MUT model and was not interpreted as altered compactness or stability (Supplementary Figure S7B). Lower raw local contact counts were observed for the selected MUT deletion-junction region than for the WT affected region (Supplementary Figure S7C). Because the two selections differed in residue and atom composition and the counts were not normalized, this observation was not interpreted as a quantitative reduction in inter-chain interaction strength. Chain-specific Cα RMSF profiles showed visually apparent differences between the WT and MUT models (Supplementary Figure S7D). Overall, these findings were considered descriptive and hypothesis-generating. To further assess the reproducibility of these short-timescale observations, three independently initialized 20-ns trajectories were additionally generated for each WT and MUT model. The common-core backbone RMSD trajectories showed broadly similar ranges across independent replicates. However, RMSD values varied across the prespecified 5–10, 10–15, and 15–20 ns intervals within individual trajectories, indicating limited short-timescale sampling consistency. Matched α3(IV) Cα RMSF profiles were likewise generally similar across replicates. The mean MUT RMSF profile was modestly higher than the WT profile at 39 of 54 matched positions, with a small segment-wide mean difference of 0.023 nm. These extended analyses are presented in the Supplementary Material and were not interpreted as evidence of full convergence or as quantitative validation of the descriptive 5-ns trajectories. Even with the extended 20-ns simulations, the timescale remains too short to faithfully model the slow folding and assembly dynamics of the collagen IV triple helix. None of the MD observations were used as independent ACMG/AMP evidence.

### 3.7 Transcript-level evidence and variant classification of *COL4A3* c.3418 + 1G>T

In the initial whole-exome sequencing report, *COL4A3* c.3418 + 1G>T was classified as a Variant of Uncertain Significance based on PVS1_Moderate, PM2_Supporting, and PP4 (Supplementary Table S2). The minigene assays subsequently demonstrated complete exon 39 skipping in HEK293T, HK-2, and HGEC cells, producing an 81-bp in-frame deletion corresponding to p. Lys1114_Gly1140del (Δ27 aa). In accordance with the 2023 ClinGen SVI splicing recommendations, the experimentally observed RNA consequence was evaluated using PVS1(RNA), rather than by applying PS3 separately (Walker et al., 2023).

Under the PVS1 decision framework, an in-frame splicing event that alters a protein region critical to function may support Strong strength, whereas an event involving a region of uncertain functional importance and removing less than 10% of the protein generally supports Moderate strength (Abou Tayoun et al., 2018). The deleted 27-amino-acid segment represents approximately 1.6% of the 1,670-amino-acid α3(IV) chain; therefore, the generic size-based pathway alone would support PVS1_Moderate. However, this segment lies within the collagenous domain, contains nine regularly spaced glycine residues, and is located between mapped natural interruptions XIX and XX of the α3(IV) chain. The Alport syndrome-specific guidance recognizes most glycine residues in the collagenous domains of the α3(IV), α4(IV), and α5(IV) chains as critical residues equivalent to a functional domain (Savige et al., 2021). On this disease-specific basis, PVS1_Strong (RNA) was considered as a conditional author-level alternative; however, because extension of the critical-region pathway to this in-frame regional deletion remains interpretive, PVS1_Moderate was adopted as the primary conservative assignment. The consistent results across 3 cell lines increased confidence in the observed RNA consequence but were not counted as an additional independent ACMG/AMP criterion.

PVS1(RNA) was not assigned at very strong strength because the event preserves the reading frame and neither an exon 39-specific expert-panel determination nor direct protein-level functional evidence has established that deletion of this segment produces a complete loss of protein function. PVS1_Moderate was therefore adopted as the primary conservative assignment, particularly because patient-derived native RNA was unavailable. PM2_Supporting was retained from the initial clinical report. PP4 was not applied in the primary classification because isolated microscopic hematuria has limited phenotypic specificity and the proband’s mother had the same phenotype but tested negative for c.3418 + 1G>T; PP4 was considered only in the conditional alternative. Accordingly, PVS1_Moderate together with PM2_Supporting yields a primary classification of Variant of Uncertain Significance. A Likely Pathogenic classification is reached only under the conditional author-level alternative in which PVS1_Strong (RNA), PM2_Supporting, and PP4 are combined. Structural modeling and exploratory molecular dynamics findings were not assigned independent ACMG/AMP evidence codes.

## 4 Discussion

Splice-site variants represent an important category of pathogenic variants in *COL4A3*, yet the transcript-level consequences of many canonical splice-site variants remain experimentally undefined (Chen et al., 2025; Daga et al., 2025). To our knowledge, this study provides the first minigene-based experimental characterization that *COL4A3* c.3418 + 1G>T causes complete exon 39 skipping and an in-frame deletion within the collagenous domain of the α3(IV) chain (Figure 5). These findings provide minigene-based transcript-level functional evidence and support transcript-level functional characterization of *COL4A3* c.3418 + 1G>T, while the primary conservative author-level classification remains VUS and Likely Pathogenic is retained only as a conditional alternative under the combined ACMG/AMP, Alport syndrome-specific, and ClinGen SVI splicing frameworks, and the structural analyses provide only hypothesis-generating context for a potential relationship between abnormal RNA splicing and the mild *COL4A3*-related phenotype (van der Loop et al., 2000; Li et al., 2018; Walker et al., 2023).

**FIGURE 5:**
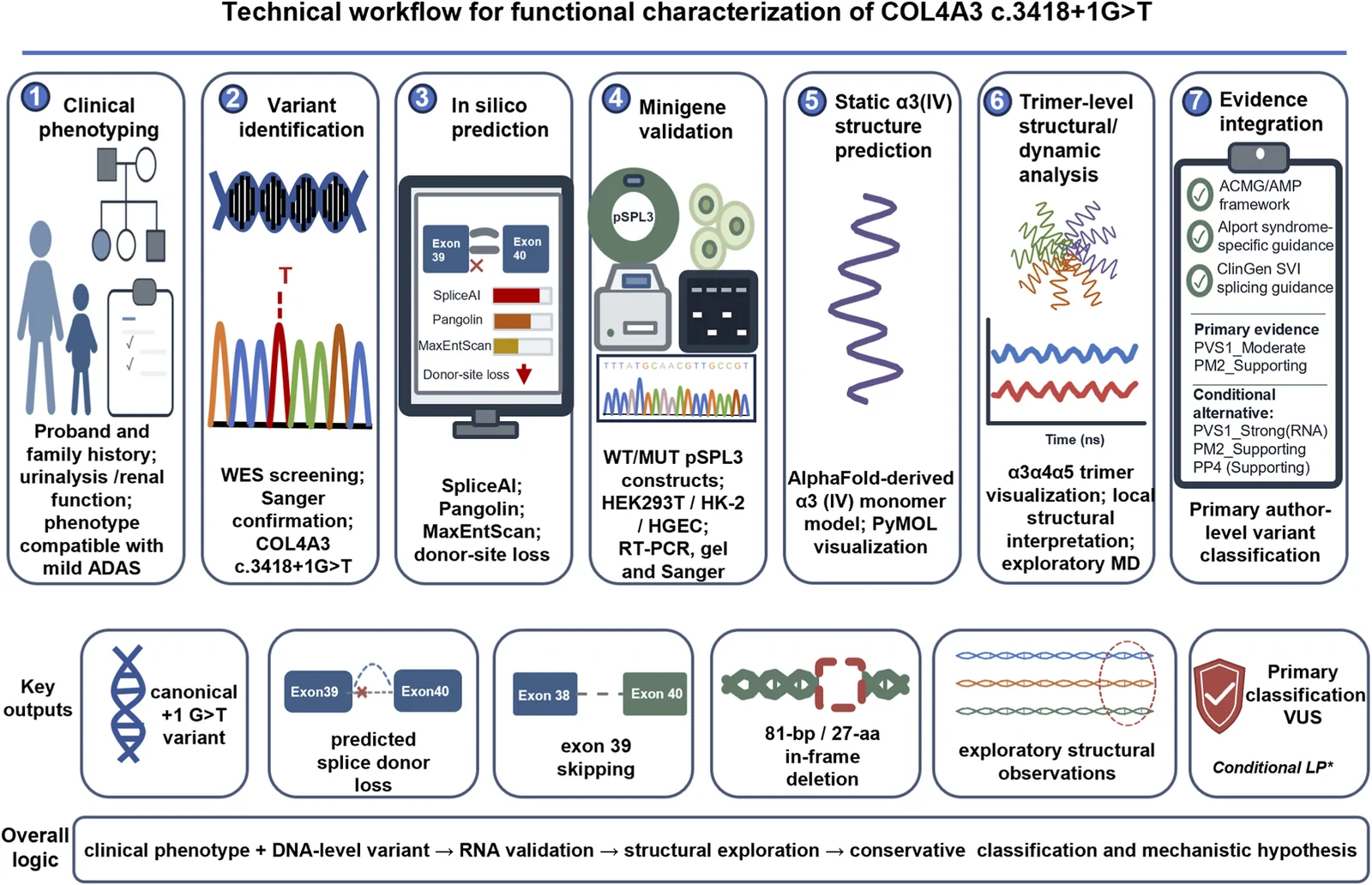
Integrated workflow for functional characterization and conservative author-level classification of *COL4A3* c.3418 + 1G>T. The study workflow integrates clinical phenotyping, variant identification, *in silico* splice prediction, minigene-based RNA validation, static α3(IV) structural prediction, trimer-level exploratory structural/dynamic analysis, and evidence integration. The key outputs connect the DNA-level canonical +1 G>T variant to predicted donor-site loss, experimentally confirmed exon 39 skipping, an 81-bp in-frame deletion predicted to remove 27 amino acids, hypothesis-generating structural observations, and a conservative author-level variant classification. Evidence was evaluated using the ACMG/AMP, Alport-specific, and ClinGen SVI splicing guidance. Under the primary conservative author-level assessment, PVS1_Moderate together with PM2_Supporting yields a Variant of Uncertain Significance (VUS) classification. A Likely Pathogenic classification is retained only as a conditional author-level alternative if PVS1(RNA) is calibrated to Strong and PP4 is applied. * LP, Likely Pathogenic. This author-level assessment does not replace an accredited clinical laboratory report.

Canonical splice-site variants affecting the invariant GT donor dinucleotide are generally predicted to disrupt normal pre-mRNA splicing (Richards et al., 2015; Abou Tayoun et al., 2018). However, computational algorithms alone cannot determine the actual transcript outcome. Therefore, experimental validation remains essential for accurate variant classification (Brnich et al., 2019). The selection of cell models was intended to evaluate the qualitative robustness of the reporter-defined splicing outcome rather than to treat the three models as equivalent physiological surrogates. HEK293 T cells were used as a technically tractable, high-transfection reference platform for the pSPL3 assay. HK-2 proximal tubular epithelial cells and human glomerular endothelial cells were included to expose the same WT and MUT constructs to two additional human renal cell lineages with potentially different trans-acting splicing environments. Because minigene splicing outcomes may depend on the cell type and reporter promoter used, concordance across different cellular backgrounds can help assess whether a qualitative splice outcome is dependent on a single assay system (Walker et al., 2023). We did not profile the abundance or activity of individual splicing factors in these cells and therefore do not attribute the observed outcome to any specific trans-acting factor. Importantly, neither HK-2 cells nor glomerular endothelial cells reproduce the podocyte context from which the glomerular α3α4α5(IV) collagen network originates (Abrahamson et al., 2009). Thus, the identical complete exon 39-skipping pattern across the three tested cell models supports the reproducibility of this qualitative result across the tested cellular backgrounds and is consistent with loss of the invariant +1 donor site being the principal cis-acting determinant within these reporter systems. However, it does not establish the precise endogenous splicing outcome in patient podocytes.

Unlike many pathogenic splice variants that generate premature termination codons through frameshifts, exon 39 skipping preserved the translational reading frame while deleting 27 amino acids from the collagenous domain. Consistent with the in-frame nature of the splice product, exploratory RT-qPCR did not show a consistent marked depletion of MUT minigene-derived transcripts across the three cellular backgrounds (Supplementary Figure S6); however, this assay assessed steady-state transcript abundance only and does not directly establish RNA stability or NMD activity. Previous studies of *COL4A3* splice variants have also shown that some splicing abnormalities can result in in-frame deletions rather than premature termination codons (van der Loop et al., 2000; Daga et al., 2025). This distinction is biologically important because the collagenous domain of type IV collagen is largely composed of Gly-X-Y repeats but contains multiple naturally occurring interruptions, which confer local flexibility and are thought to contribute to collagen IV network assembly (Mariyama et al., 1994; Hudson et al., 2003). Sequence-level examination showed that p. Lys1114_Gly1140del did not shift the Gly-X-Y register. In wild-type numbering, Gly1113 was joined to Leu1141-Pro1142-Gly1143; after mutant renumbering, the resulting sequence was Gly1113-Leu1114-Pro1115-Gly1116, thereby retaining a Gly-X-Y-Gly pattern with glycine residues spaced at every third position. Thus, the potential structural consequences of this deletion should not be attributed to disruption of the primary-sequence glycine periodicity. Nevertheless, preservation of the Gly-X-Y register does not establish preservation of normal protein structure. Formation of collagen IV trimers depends on specific recognition and assembly among alpha chains, and the NC1 domain plays an important role in chain selection and triple-helical protomer formation (Hudson et al., 2003; Khoshnoodi et al., 2006). In this study, AlphaFold structural prediction was performed separately using the wild-type α3(IV) sequence and the mutant α3(IV) sequence generated after exon 39 skipping, followed by visualization and comparison in PyMOL. These models were used to visualize the location of the deleted region and the newly juxtaposed sequence in the α3(IV) monomer and α3α4α5(IV) trimer contexts; however, they cannot determine whether preservation of the primary-sequence Gly-X-Y register translates into normal triple-helical packing (Jumper et al., 2021; Varadi et al., 2022). If the mutant α3(IV) chain is translated and incorporated into the α3α4α5(IV) heterotrimer, shortening of the α3(IV) chain by 27 amino acids and juxtaposition of previously separated sequence regions may affect local interchain alignment, triple-helical packing, secretion, or heterotrimer assembly. These possibilities remain hypothetical because protein-level expression and assembly were not directly examined. Future studies could compare wild-type and mutant α3(IV) chains in recombinant α3α4α5(IV) expression systems using secretion and heterotrimer-assembly assays. Junction-spanning collagen-like peptides could also be evaluated by circular-dichroism thermal analysis to examine their intrinsic ability to form and maintain a local triple-helical conformation. The descriptive analysis presented in Supplementary Figure S7 was based on one 5-ns trajectory for each local trimer model and showed differences in RMSD, Rg, RMSF, and raw local contact-count profiles. The contact analysis compared selections that differed in residue and atom composition and was not normalized; therefore, the lower raw counts in MUT cannot be interpreted as a quantitative reduction in interaction strength. To further assess the reproducibility of these exploratory observations, we additionally generated three independently initialized 20-ns trajectories for each model. The extended simulations showed broadly similar common-core RMSD ranges and generally similar matched α3(IV) Cα RMSF profiles across independent replicates. However, RMSD values varied across prespecified time blocks within individual trajectories, indicating limited short-timescale sampling consistency. The mean MUT α3(IV) RMSF profile was only modestly higher than the WT profile across the matched segment. Accordingly, the additional 20-ns simulations were interpreted as a supplementary reproducibility assessment rather than as validation of the 5-ns observations or evidence of full convergence. Moreover, even the extended 20-ns simulations remain too short to faithfully model the slow folding and assembly dynamics of the collagen IV triple helix and were not used as ACMG/AMP classifiable evidence. Neither the supplementary 5-ns analysis nor the 20-ns replicate simulations demonstrate altered protein stability or impaired α3α4α5(IV) heterotrimer *assembly in vivo;* rather, they provide hypothesis-generating structural observations for future experimental testing. Studies in *COL4A3*-deficient animal models have also shown that abnormalities of the α3(IV) chain may be accompanied by absent or abnormal distribution of α4(IV) and α5(IV) chains in the GBM, thereby affecting GBM structural stability (Cosgrove et al., 1996; Miner and Sanes, 1996).

An additional observation of clinical relevance is the patient’s relatively mild renal phenotype; however, its relationship to c.3418 + 1G>T cannot be established from the present data. The patient presented with persistent isolated microscopic hematuria without proteinuria, renal insufficiency, hearing impairment, or ocular abnormalities, a presentation compatible with the mild end of the heterozygous *COL4A3*-related disease spectrum (Kashtan et al., 2018; Savige et al., 2022). Previous studies have shown that *COL4A3*/*COL4A4*-related nephropathy may represent a phenotypic continuum ranging from benign familial hematuria to autosomal dominant or autosomal recessive Alport syndrome (Longo et al., 2002). Patients with autosomal recessive Alport syndrome usually carry biallelic pathogenic variants in *COL4A3* or *COL4A4* and generally exhibit a more severe phenotype than heterozygous individuals (Mochizuki et al., 1994; Storey et al., 2013). The in-frame splice outcome does not by itself demonstrate preservation of partial collagen IV function, because protein expression, secretion, α3α4α5(IV) heterotrimer assembly, and basement-membrane incorporation were not directly examined. Li et al. (2018) previously reported *COL4A3* c.3418 + 1G>T in cis with *COL4A4* c.1471C>T in a large Chinese family, in which the two variants co-segregated with a phenotype described as benign familial hematuria. By contrast, *COL4A4* c.1471C>T was not detected in the proband’s WES data. The in-cis configuration in the previous family makes it difficult to distinguish the independent contribution of either variant, whereas the uninformative segregation data in the present family also preclude attributing the phenotype solely to c.3418 + 1G>T. Clinically, the two reports are more similar than distinct, because both include persistent hematuria with preserved renal function; the different descriptions of benign familial hematuria and mild autosomal dominant Alport syndrome may therefore partly reflect differences in ascertainment, diagnostic terminology, age, and follow-up duration. Differences in genetic background, modifying variants, and non-genetic factors may also contribute. In addition, the mother had microscopic hematuria but did not carry c.3418 + 1G>T, whereas paternal renal evaluation and genetic testing were unavailable; consequently, neither informative co-segregation nor a *de novo* occurrence could be established. Alternative genetic or non-genetic explanations for the hematuria in the patient and her mother therefore cannot be excluded. More generally, heterozygous *COL4A3*-related disease encompasses a broad phenotypic spectrum ranging from isolated hematuria to progressive chronic kidney disease (Kashtan et al., 2018; Savige et al., 2022). In addition, early genetic studies of *COL4A5* and *COL4A3*/*COL4A4* established the genetic classification of Alport syndrome and indicated that the affected gene, variant type, and allelic status may all influence clinical phenotype (Barker et al., 1990; Mochizuki et al., 1994; Bekheirnia et al., 2010). In X-linked Alport syndrome, variant type and location have been shown to correlate with renal, auditory, and ocular phenotypes, further supporting the need for genotype–phenotype analyses in *COL4A3*-related disease (Bekheirnia et al., 2010; Zhang et al., 2018).

Our study also illustrates the value of integrating transcript-level assays into clinical variant classification. Current ACMG/AMP guidelines recognize functional evidence as important for variant classification (Richards et al., 2015), and subsequent recommendations emphasize that evidence strength should consider assay quality, disease mechanism, and the molecular consequence of the variant (Abou Tayoun et al., 2018; Brnich et al., 2019). More specifically, the 2023 ClinGen SVI splicing recommendations advise capturing experimentally observed loss-of-function RNA outcomes using PVS1_Strength (RNA) rather than applying PS3 separately to the same splicing result (Walker et al., 2023). When patient RNA is unavailable, minigene assays provide a practical approach for defining transcript consequences, whereas structural prediction and visualization can provide additional, non-classifying mechanistic clues regarding how an abnormal transcript may affect protein architecture (Liang et al., 2023; Chen et al., 2025). Together, these approaches provide substantially more information than computational prediction alone, although they do not all constitute independent ACMG/AMP criteria. Large-scale exome sequencing studies have also highlighted the diagnostic utility of genetic testing in kidney diseases, particularly for identifying inherited kidney disorders that are difficult to define by conventional clinical or pathological evaluation (Groopman et al., 2019). Because early identification of patients with Alport syndrome can inform follow-up management and kidney-protective treatment, clarifying the functional consequence of splice-site variants has potential clinical significance (Gross et al., 2012). In addition, increasing evidence suggests that coding variants may also affect splicing, indicating that sequence variants should not be interpreted solely according to their predicted protein-level consequences at the DNA level (Soemedi et al., 2017).

In our author-level application of these frameworks, the experimentally observed complete exon 39 skipping was evaluated using PVS1(RNA), with PVS1_Moderate adopted as the primary assignment, without separately applying PS3 to the same RNA evidence. The 81-bp event preserves the reading frame and removes 27 amino acids, corresponding to approximately 1.6% of the 1,670-amino-acid α3(IV) chain; therefore, the deletion-size pathway alone would support Moderate strength. We conditionally calibrated PVS1(RNA) to Strong through the critical-region pathway because the deleted segment lies within the collagenous domain, between the mapped natural interruptions XIX (residues 1,060–1,064) and XX (residues 1,176–1,179), and contains nine regularly spaced glycine residues. Very Strong strength was not assigned because the transcript remains in frame, is not expected to undergo nonsense-mediated decay, and direct protein-level functional evidence was unavailable. PVS1_Moderate was therefore adopted as the primary conservative assignment in the absence of patient-derived RNA and an exon 39-specific expert-panel rule. PM2_Supporting was retained from the initial clinical report, which documented absence of the variant from the population databases evaluated at that time, whereas PP4 was not applied in the primary classification because the phenotype has limited specificity, but was considered as Supporting evidence only in the conditional alternative. The primary evidence combination of PVS1_Moderate and PM2_Supporting yields a Variant of Uncertain Significance classification. A Likely Pathogenic classification is reached only under the conditional author-level alternative combining PVS1_Strong (RNA), PM2_Supporting, and PP4 (Supporting) (Supplementary Table S2).

Several limitations should be acknowledged. First, all transcript-level evidence in this study was obtained using heterologous pSPL3 minigene reporter assays rather than endogenous RNA isolated from the patient. Although complete exon 39 skipping was reproduced in HEK293T, HK-2, and HGEC models, this concordance demonstrates reproducibility only across the tested reporter systems and does not constitute definitive *in vivo* transcript validation. Moreover, the abundance and activity of splicing factors were not directly profiled in these cells, and none of the tested models reproduces the native *COL4A3*-expressing podocyte splicing environment. The pSPL3 construct contains only exon 39 and limited flanking intronic sequences; consequently, it cannot fully reproduce the endogenous *COL4A3* chromatin and transcriptional context and may omit distant cis-regulatory elements, including splicing enhancers or silencers located outside the cloned fragment. Confirmation using patient-derived endogenous RNA would ultimately be required if an appropriate clinical sample becomes available (Chen et al., 2025; Daga et al., 2025). Second, structural analysis was based on AlphaFold-predicted models and PyMOL visualization, and the molecular dynamics simulations used AlphaFold Server-derived local α3α4α5(IV) trimer starting structures. The descriptive analysis presented in Supplementary Figure S7 was based on one 5-ns trajectory for each model and was not intended as a replicate-level production simulation study. To further assess reproducibility, we additionally generated three independently initialized 20-ns trajectories for each WT and MUT model and examined replicate-level RMSD behavior, matched α3(IV) RMSF profiles, and prespecified 5–10, 10–15, and 15–20 ns RMSD summaries in the Supplementary Material. These extended simulations showed broadly similar RMSD ranges across independent replicates, although variation in RMSD across prespecified time blocks indicated limited short-timescale sampling consistency. Importantly, even a 20-ns timescale remains insufficient to capture the slow folding and assembly dynamics of the collagen IV triple helix. In addition, the raw local contact counts in the 5-ns analysis were not normalized for differences in residue and atom composition between the WT affected-region and MUT deletion-junction selections. Accordingly, neither the supplementary 5-ns analysis nor the 20-ns simulations provide evidence of full convergence, quantitative differences in interaction strength or protein stability, or impaired α3α4α5(IV) heterotrimer assembly *in vivo*. The MD findings should therefore be regarded as exploratory and hypothesis-generating (Jumper et al., 2021; Varadi et al., 2022). Third, the primary VUS classification reflects a conservative author-level assessment, whereas the alternative Likely Pathogenic classification remains conditional on the applicability of PVS1_Strong (RNA) and PP4 and does not replace an official clinical laboratory report. Finally, this study investigated a single family. The mother’s microscopic hematuria did not co-segregate with c.3418 + 1G>T. Although the father was reported to have no known renal manifestations, this could not be confirmed because contemporaneous urinalysis, renal function measurements, other formal renal evaluations, and paternal genetic testing were unavailable. Therefore, neither co-segregation nor a *de novo* origin could be established from the available family data. The available family data cannot attribute the patient’s hematuria solely to c.3418 + 1G>T, and alternative genetic or non-genetic explanations for the hematuria in the patient and her mother cannot be excluded. Additional clinical follow-up and independent functional or clinical observations will be needed to refine the genotype–phenotype relationship (van der Loop et al., 2000; Longo et al., 2002; Kashtan et al., 2018).

## 5 Conclusion

The canonical *COL4A3* splice-site variant c.3418 + 1G>T abolishes normal donor splice-site recognition and induces complete exon 39 skipping in minigene assays, resulting in an 81-bp/27-amino-acid in-frame deletion within the collagenous domain of the α3(IV) chain. Minigene-based functional validation provides experimental evidence for the observed splicing consequence of this variant, whereas structural visualization and the exploratory molecular dynamics analyses—including the supplementary 5-ns and 20-ns analyses—provide only hypothesis-generating context for potential protein-level effects and do not establish altered protein stability or impaired α3α4α5(IV) heterotrimer assembly. Under the specified ACMG/AMP evidence framework, the primary conservative classification is Variant of Uncertain Significance based on PVS1_Moderate and PM2_Supporting; Likely Pathogenic is retained only as a conditional author-level alternative if PVS1(RNA) is calibrated to Strong and PP4 is applied. This conditional author-level assessment does not replace an official clinical laboratory report. The study highlights the importance of experimentally defining RNA consequences when interpreting canonical splice-site variants. Patient-derived RNA, informative family segregation, and direct protein-level studies are required before firm genotype–phenotype or mechanistic conclusions can be drawn.

## Data Availability

The datasets presented in this study can be found in online repositories. The names of the repository/repositories and accession number(s) can be found below: https://figshare.com/, 10.6084/m9.figshare.33941410 https://www.ncbi.nlm.nih.gov/, PRJNA1531752.
