# Supplementary material for: The COL4A3 c.3418+1G>T variant causes exon 39 skipping in minigene assays: functional and exploratory structural analyses
Source: Front Genet. 2026 Oct 13;17:1953498. doi: 10.3389/fgene.2026.1953498 (PMC13706678; doi:10.3389/fgene.2026.1953498)
Supplement: Supplementary file 1 [file DataSheet1.pdf]

Supplementary Material

1 Supplementary Methods

Supplementary Method S1. Construction of local α3α4α5(IV) trimer models and exploratory molecular dynamics analysis

The WT and MUT local α3α4α5(IV) trimer models were generated using AlphaFold Server from sequence windows centered on the exon 39-encoded region. Chain A, chain B, and chain C represented α3(IV), α4(IV), and α5(IV), respectively. The WT model contained 81 amino acids in each chain. In the MUT model, deletion of the exon 39-encoded 27 amino acids shortened chain A to 54 amino acids, whereas chains B and C remained 81 amino acids long. The sequence windows were selected according to Gly-X-Y periodicity; this procedure was used for local model construction and was not considered validation of a unique biological collagen-chain register.

Simulations were performed using GROMACS 2025.4 (CUDA) with the charmm36-jul2022.ff CHARMM36 July 2022 GROMACS distribution; its protein parameters are the CHARMM36m/all36m set, and the accompanying CHARMM TIP3P water model (tip3p.itp) was used. Each model was placed in a principal-axis-aligned rectangular box with nominal 1.0-nm padding, solvated, adjusted to approximately 0.15 M NaCl, and neutralized. Energy minimization was performed using the steepest-descent algorithm until the maximum force was <1,000 kJ mol−1 nm−1. This was followed by 50-ps NVT equilibration at 310 K and 50-ps NPT equilibration at 310 K and 1 bar with position restraints. Temperature was controlled using V-rescale coupling, and pressure was controlled using semi-isotropic C-rescale coupling. Each trajectory used for Supplementary Figure S7 comprised a 1-ns production segment followed by a 4-ns continuation from the same checkpoint. Production MD used a 2-fs time step, particle-mesh Ewald electrostatics, 1.2-nm Coulomb and van der Waals cutoffs, LINCS constraints for bonds involving hydrogen atoms, and periodic boundary conditions in all three dimensions.

Trajectories were processed using PBC nojump correction, protein centering, and backbone fitting. Backbone RMSD was calculated relative to the processed 0-ns reference frame, Rg was calculated for the entire local trimer, and RMSF was calculated separately for chain-specific Cα selections. The WT local-contact selection comprised chain A residues 28–54, corresponding to α3(IV) residues 1114–1140. The MUT selection comprised chain A residues 22–33, corresponding to residues 1108–1113 and 1141–1146 surrounding the deletion junction. Heavy-atom contacts within 0.5 nm with chains B and C were calculated separately and summed. Because the WT and MUT selections differed in residue and atom composition, the raw counts were not normalized and were not interpreted as binding energies or quantitative measures of interaction strength. All four panels of Supplementary Figure S7 were derived from the same WT and MUT main trajectory pair. To further assess the reproducibility of the exploratory short-timescale observations, three independently initialized 20-ns production trajectories were additionally generated for each WT and MUT local trimer model using the same force field, solvent model, ionic conditions, equilibration procedure, temperature, pressure, and production settings described above. Independent initialization was achieved using different velocity seeds for each replicate, followed by separate NVT/NPT equilibration and production runs. For the replicate analyses, each raw trajectory was first reconstructed using PBC clustering and centering to maintain the three-chain α3α4α5(IV) trimer as a continuous assembly across periodic boundaries. The reconstructed trajectory was then fitted by rotation and translation to its processed frame-0 reference using a sequence-matched 861-atom common structural core that was identical in atom identity and order

between WT and MUT models. The same common-core selection was used for backbone RMSD calculation.

For α3(IV) RMSF analysis, Cα RMSF was calculated separately for each fitted trajectory using 54 homologous α3(IV) Cα atoms shared between the WT and MUT models. The matched positions comprised WT residues 1–25 and 53–81, mapped in order to MUT residues 1–54; the alignment gap spans WT local residues 26–52, an alternative placement that yields the same MUT sequence as deletion of residues 28–54. Mean RMSD values were additionally summarized over prespecified 5–10, 10–15, and 15–20 ns intervals for each individual trajectory to assess short-timescale sampling consistency. These analyses were used as supplementary reproducibility assessments and were not interpreted as evidence of full convergence or as quantitative validation of the 5-ns simulations shown in Supplementary Figure S7. No inferential statistical testing was performed using individual MD frames. Because even a 20-ns simulation remains short relative to the slow folding and assembly dynamics of collagen IV triple helices, the replicate simulations were interpreted descriptively and not as a direct model of in-vivo heterotrimer folding or assembly.

2 Supplementary Figures and Tables

2.1 Supplementary Figures

Supplementary Figure S1. Sanger sequencing of the *COL4A3* c.3418+1G>T site in the proband and her mother

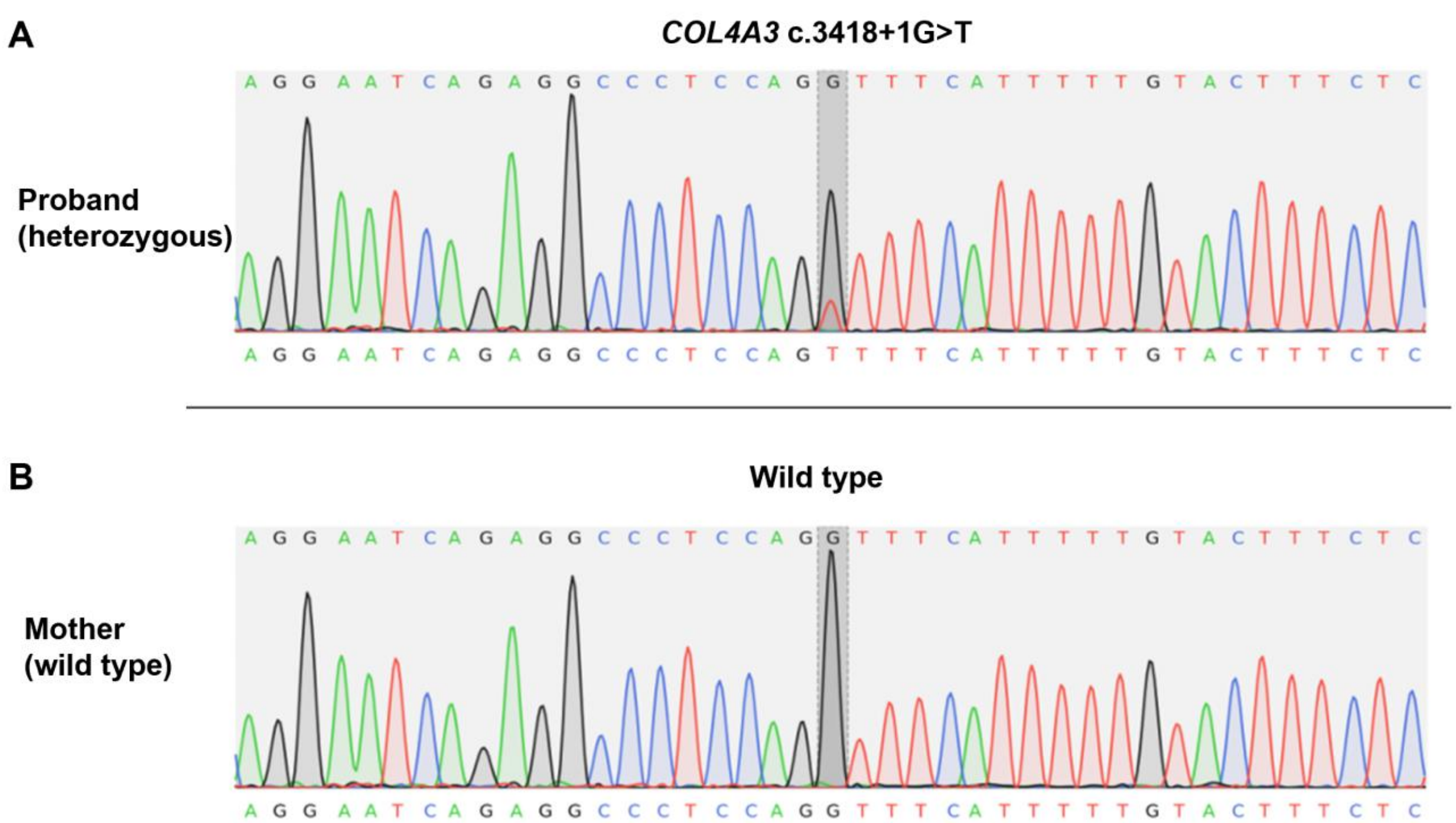

Supplementary Figure S1. Sanger sequencing of the *COL4A3* c.3418+1G>T site in the proband and her mother. (A) The proband showed a heterozygous G>T substitution at position c.3418+1. (B) The mother showed the wild-type sequence at the corresponding position. The gray-shaded regions indicate the variant position. Panel A reproduces the proband chromatogram shown in main Figure 1B to facilitate direct comparison with the maternal wild-type trace.

Supplementary Figure S2. Genomic-view splice-prediction tracks for *COL4A3* c.3418+1G>T

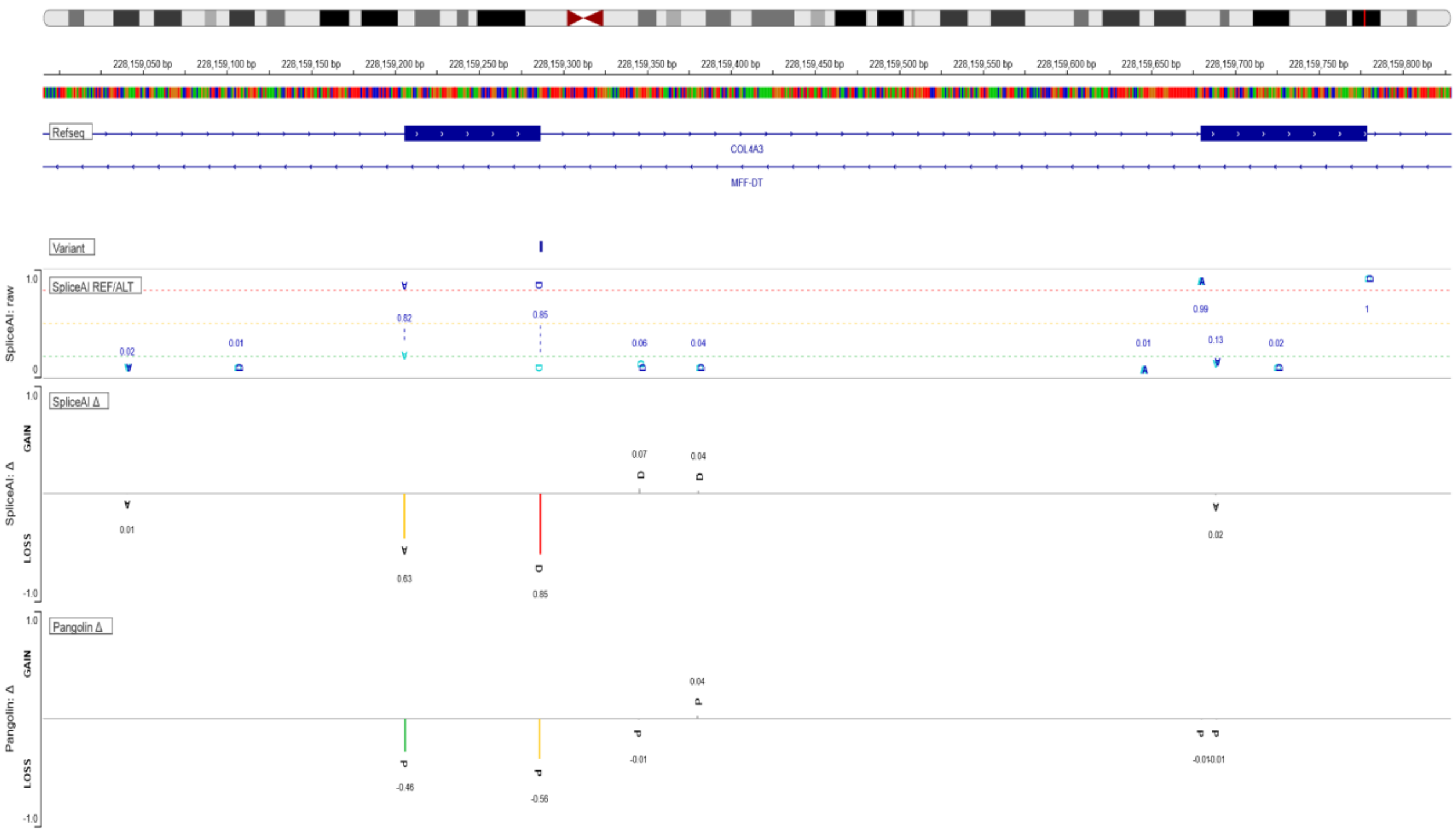

Supplementary Figure S2. Genomic-view splice-prediction tracks for *COL4A3* c.3418+1G>T. Detailed graphical outputs show the predicted effects of the reference and alternative alleles on pre-mRNA splicing. SpliceAI predicted donor loss at position −1 (Δ score = 0.85) and acceptor loss at position −81 (Δ score = 0.63), whereas Pangolin predicted splice loss at position −1 (Δ score = 0.56). Lower-scoring splice-gain signals are also displayed as part of the original prediction outputs.

Supplementary Figure S3. Detailed splice-prediction outputs from SpliceAI, Pangolin, and MaxEntScan

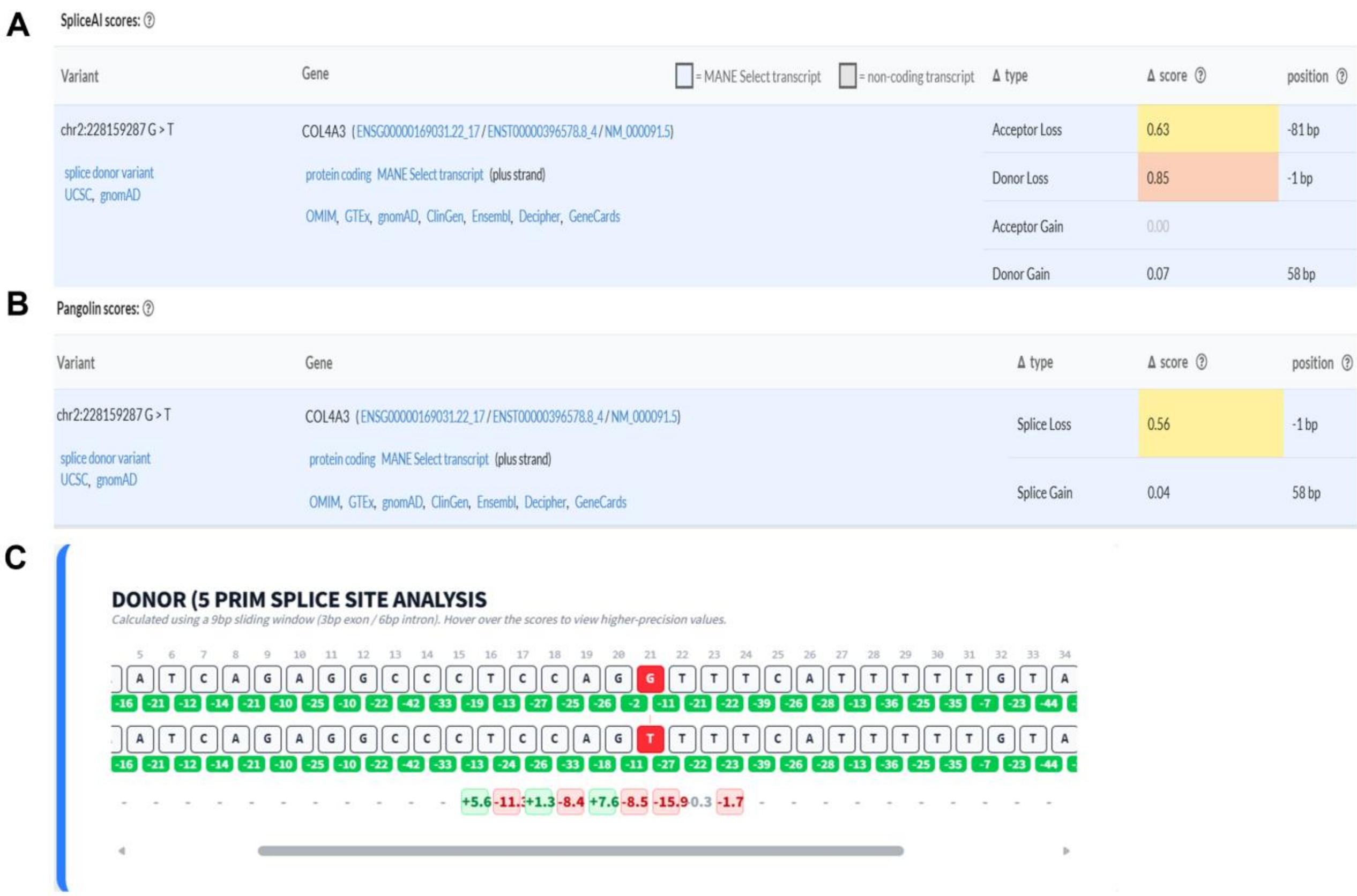

Supplementary Figure S3. Detailed splice-prediction outputs from SpliceAI, Pangolin, and MaxEntScan for *COL4A3* c.3418+1G>T. (A) SpliceAI predicted donor loss at position −1 (Δ score = 0.85), acceptor loss at position −81 (Δ score = 0.63), and a low-scoring donor-gain signal at position +58 (Δ score = 0.07). (B) Pangolin predicted splice loss at position −1 (Δ score = 0.56) and a low-scoring splice-gain signal at position +58 (Δ score = 0.04). (C) MaxEntScan analysis of the native 5′ splice donor site showed reduced donor-site strength following the G>T substitution (ΔMaxEnt score [MUT−WT] = −8.5). WT, wild type; MUT, mutant.

Supplementary Figure S4. Additional minigene splicing results in HGECs and HK-2 cells

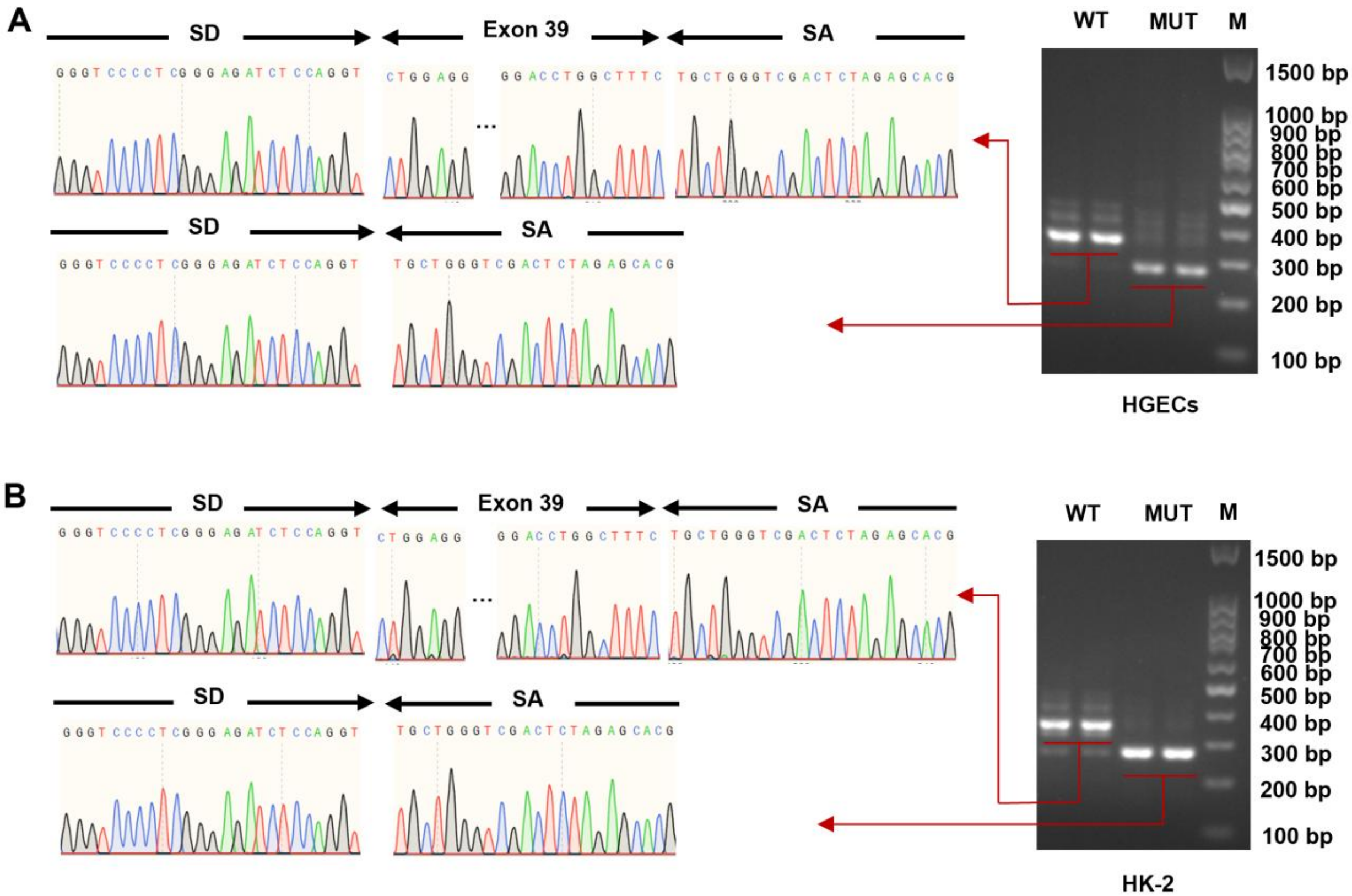

Supplementary Figure S4. Additional minigene splicing results in HGECs and HK-2 cells. (A) Results obtained in human glomerular endothelial cells (HGECs). (B) Results obtained in HK-2 cells. In both cell lines, the WT construct generated an exon 39-containing transcript, whereas the MUT construct generated a shorter transcript with direct joining of the pSPL3 SD and SA exons. Agarose gel electrophoresis showed the longer WT and shorter MUT RT-PCR products, and Sanger sequencing confirmed complete exon 39 skipping in the MUT transcript, resulting in an 81-bp deletion. WT, wild-type construct; MUT, mutant construct; M, molecular-weight marker; SD and SA, the vector-derived splice donor and splice acceptor exons, respectively. For each condition in each cell line, two independent experimental replicates are shown.

Supplementary Figure S5. Original agarose gel image of the minigene RT-PCR products

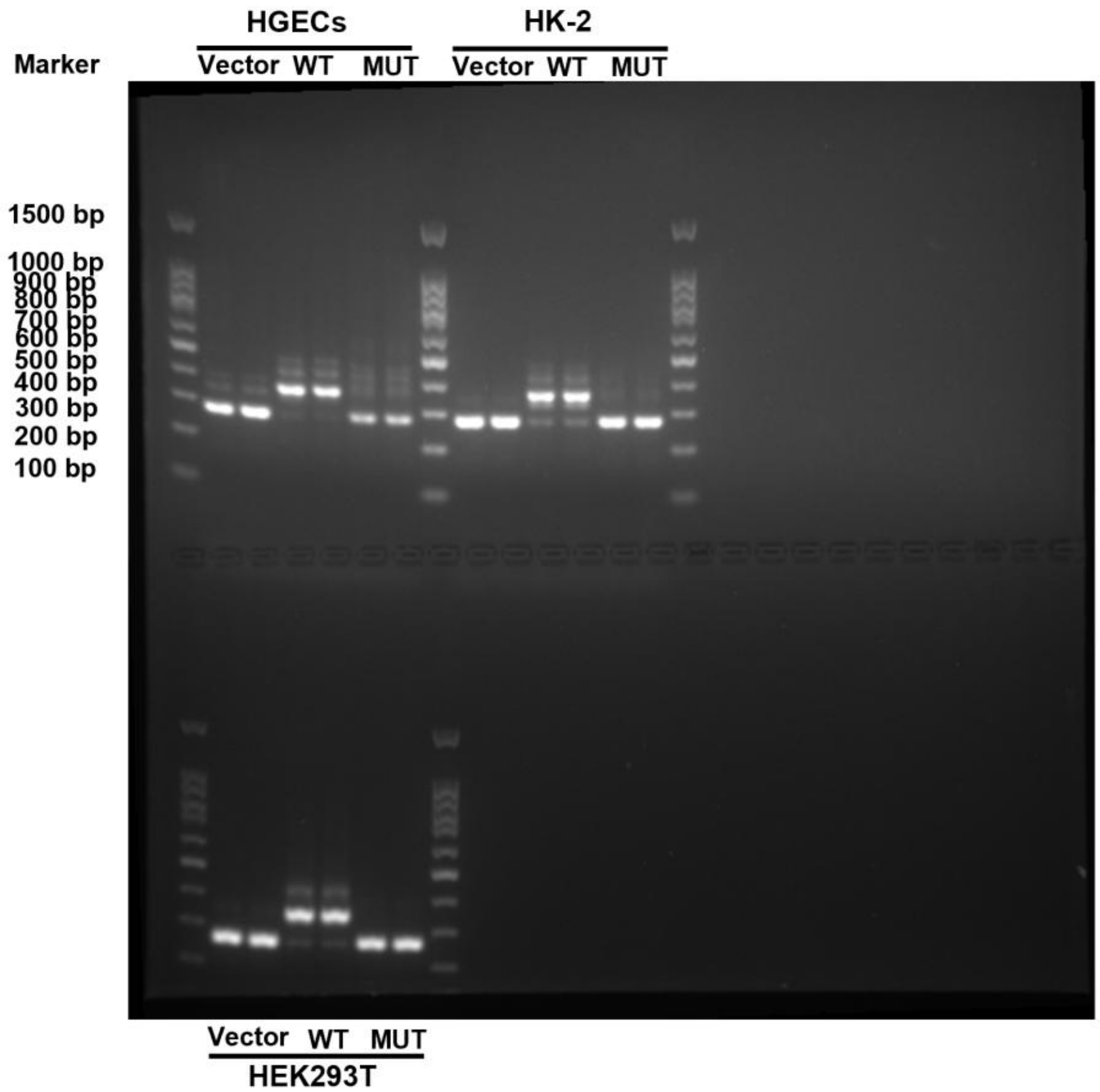

Supplementary Figure S5. Original agarose gel image of the minigene RT-PCR products. Agarose gel electrophoresis of RT-PCR products obtained from human glomerular endothelial cells (HGECs), HK-2 cells, and HEK293T cells is shown. In all three cell lines, the WT construct produced a longer product containing exon 39, whereas the MUT construct produced a shorter product consistent with complete exon 39 skipping. Adjacent lanes under the same label represent two independent experimental replicates. Vector, empty-vector control; WT, wild-type construct; MUT, mutant construct; Marker, molecular-weight marker. Lanes on the right side of the original gel were unrelated to the present study and are retained without alteration.

Supplementary Figure S6. Steady-state abundance of minigene-derived transcripts assessed by RT-qPCR

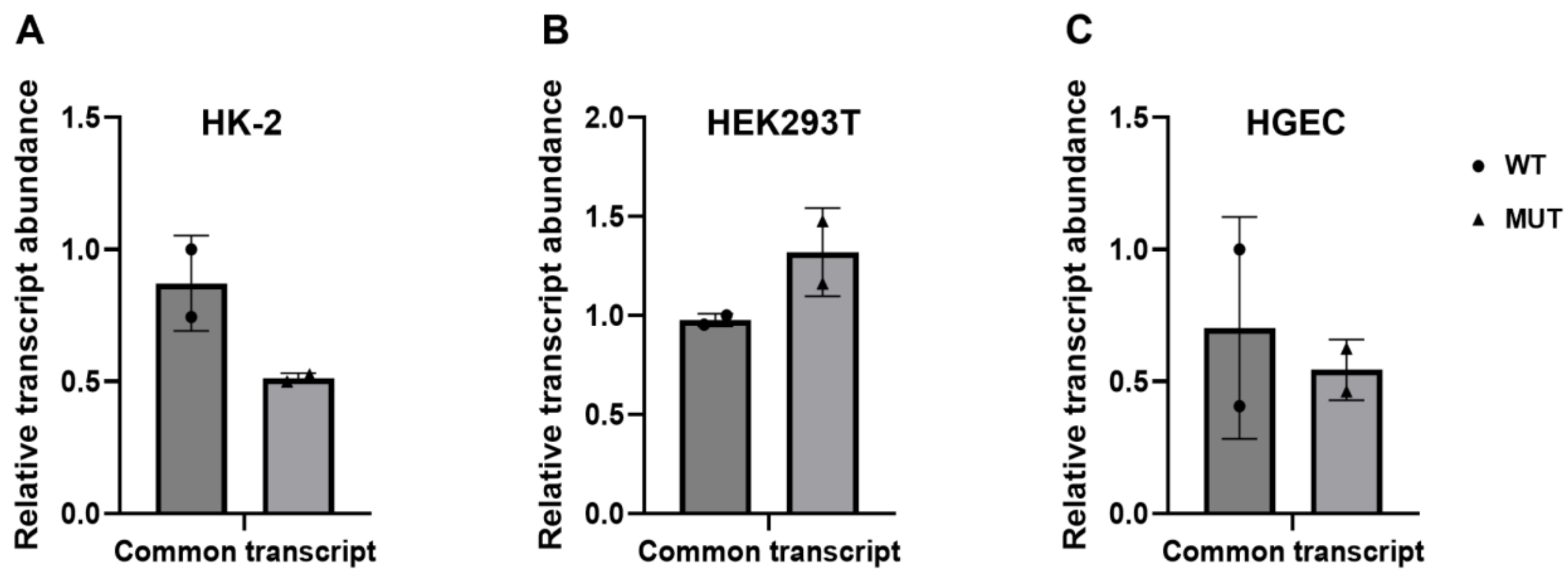

Supplementary Figure S6. Steady-state abundance of minigene-derived transcripts assessed by RT-qPCR. RT-qPCR was performed in HEK293T, HK-2, and HGEC cells using primers targeting a pSPL3-derived exonic region shared by the WT and MUT transcripts. Relative transcript abundance was normalized to ACTB. Bars represent mean ± SD, and each point represents one independent transfection experiment (n = 2 per construct in each cell line). Although some variation was observed between WT and MUT constructs, no consistent marked depletion of the MUT minigene-derived transcript was observed across the three cellular backgrounds. This assay measures steady-state transcript abundance and does not directly assess RNA half-life or nonsense-mediated decay activity. WT, wild type; MUT, mutant.

Supplementary Figure S7. Exploratory 5-ns molecular dynamics analysis of AlphaFold Server-derived WT and MUT α3α4α5(IV) local trimer models.

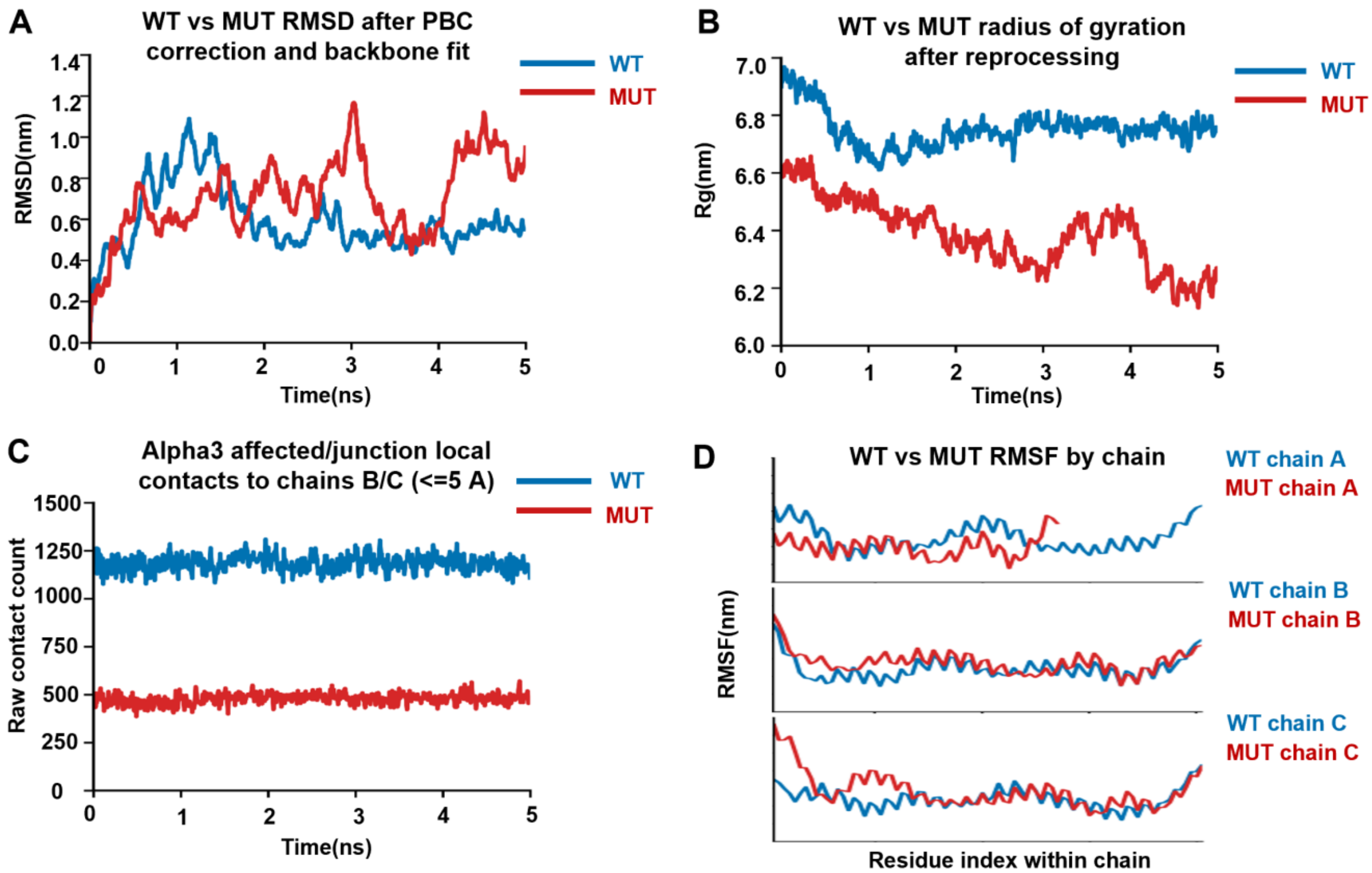

Exploratory, hypothesis-generating only; no statistical inference performed

Supplementary Figure S7. Exploratory 5-ns molecular dynamics analysis of AlphaFold Server-derived WT and MUT α3α4α5(IV) local trimer models. (A) RMSD trajectories after PBC correction, protein centering, and backbone fitting. Values represent the deviation of each model from its respective starting reference frame and were not used as a direct comparison of structural stability. (B) Radius of gyration (Rg) trajectories; differences between the WT and MUT profiles may partly reflect the shorter α3(IV) chain in the MUT model and were not interpreted as differences in compactness or stability. (C) Raw heavy-atom contact counts within 5 Å between the selected WT α3(IV) affected region or MUT α3(IV) deletion-junction region and the neighboring α4(IV)/α5(IV) chains. Because the WT and MUT selections differed in residue and atom composition, these values were not normalized and should not be interpreted as quantitative measures of interaction strength. (D) Chain-specific Cα RMSF profiles for α3(IV) (chain A), α4(IV) (chain B), and α5(IV) (chain C). The 5-ns simulations were exploratory, were based on one trajectory per model, and did not include replicate-level statistical inference. Given the short simulation timescale, these observations were not interpreted as evidence of full convergence, altered structural stability, or impaired α3α4α5(IV) heterotrimer assembly in vivo. The MD findings were not used as ACMG/AMP classifiable evidence.

Supplementary Figure S8. Individual common-core backbone RMSD trajectories from independently initialized 20-ns WT and MUT simulations.

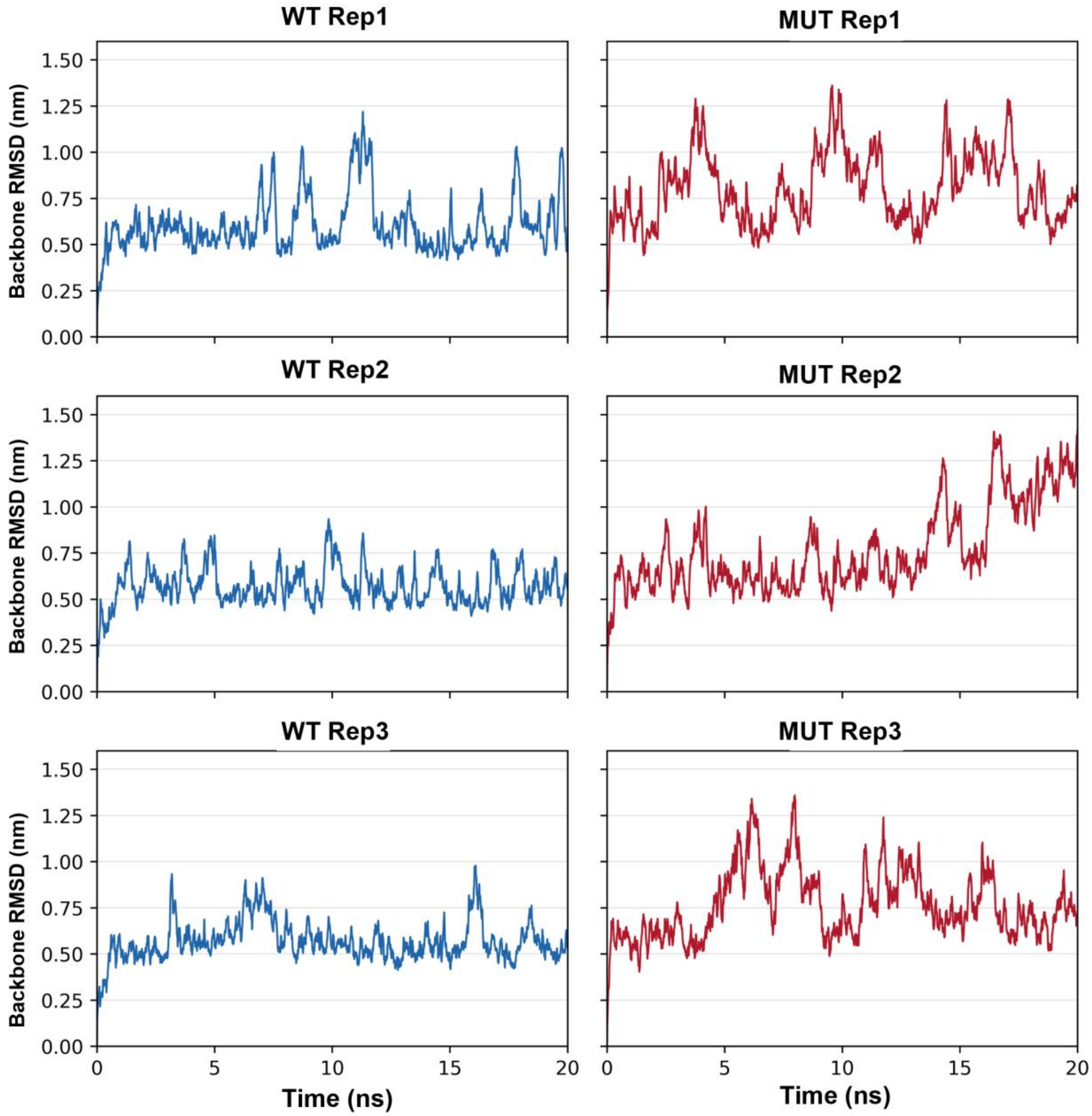

Supplementary Figure S8. Three independently initialized 20-ns trajectories were generated for each WT and MUT α3α4α5(IV) local trimer model. Before RMSD calculation, each trajectory was reconstructed to maintain the three-chain trimer as a continuous assembly across periodic boundaries and was then fitted using the sequence-matched common structural core. Rows correspond to replicates 1–3 and columns to WT and MUT models. Identical time and RMSD scales were used across panels. The trajectories showed replicate- and time-dependent variability. However, variation in RMSD across prespecified time blocks indicated limited short-timescale sampling consistency. These trajectories were not interpreted as evidence of full convergence or as a quantitative comparison of structural stability.

Supplementary Figure S9. Matched α3(IV) Cα RMSF profiles across three independent 20-ns WT and MUT simulations.

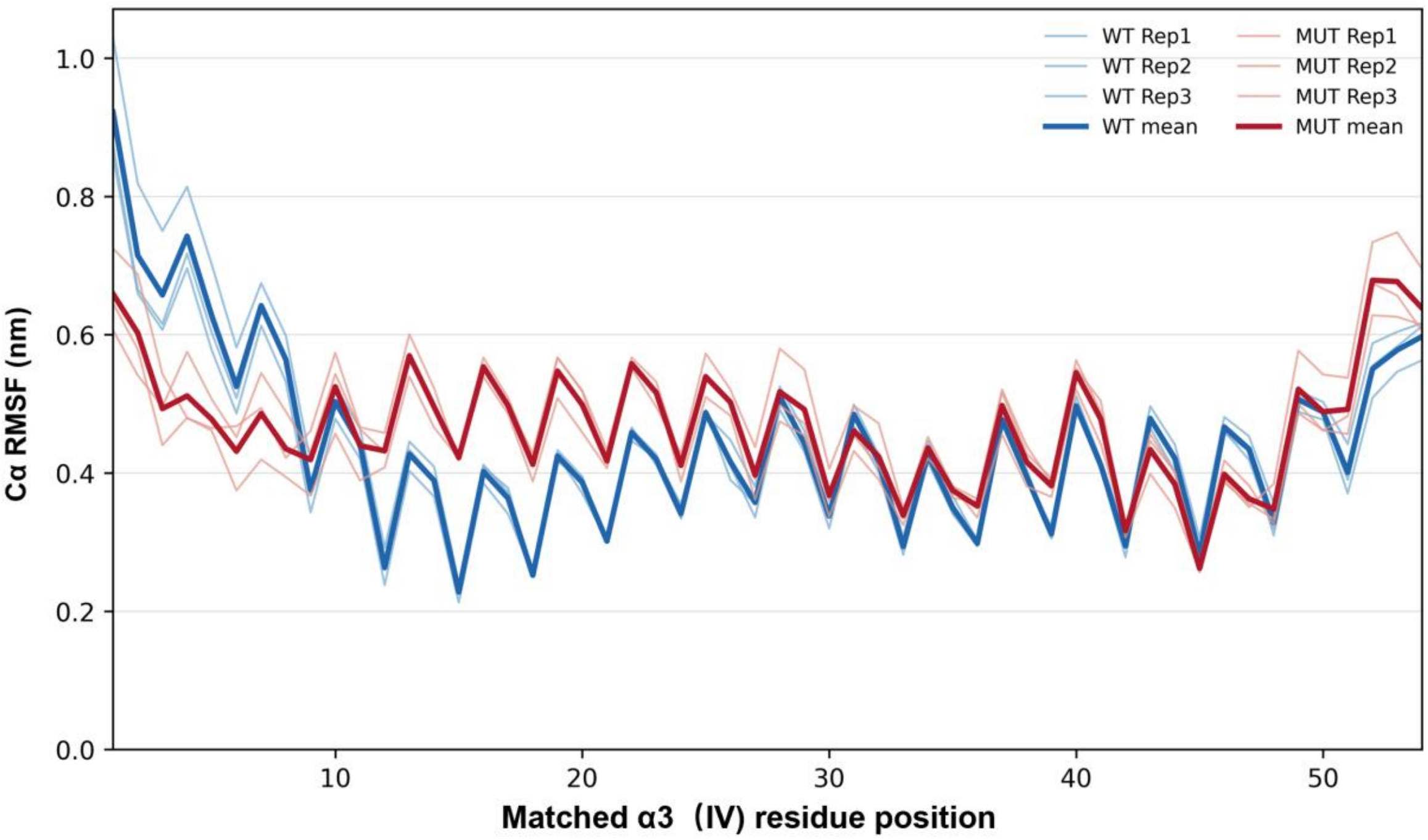

Supplementary Figure S9. Cα RMSF profiles were calculated from fitted trajectories using 54 homologous α3(IV) Cα residues shared between the WT and MUT models. For WT, residues 1–25 and 53–81 were mapped in order to MUT residues 1–54. Pale lines represent individual replicate trajectories, and dark lines represent the corresponding replicate means. RMSF profiles showed generally similar residue-wise patterns across independent replicates. The mean MUT profile was modestly higher than the WT profile at 39 of 54 matched positions, with a segment-wide mean difference of 0.023 nm. These profiles were interpreted descriptively, and no inferential statistical testing was performed using individual trajectory frames.

2.2 Supplementary Tables

Supplementary Table S1. Minigene-related primers used for RT-PCR, RT-qPCR, and sequencing

Primer	Sequence (5′–3′)	Application
pSPL3-F	GTTGTGTGGTCCATAGTAATCA	Plasmid construct verification / Sanger sequencing
pSPL3-R	ACACCGGCATGTGTGGCC	Plasmid construct verification / Sanger sequencing
SD6	TCTGAGTCACCTGGACAACC	Vector-specific minigene RT-PCR / transcript sequencing
SA2	ATCTCAGTGGTATTTGTGAGC	Vector-specific minigene RT-PCR / transcript sequencing
pSPL3-qPCR-F	AGTCACCTGGACAACCTCAAAG	Minigene-derived transcript RT-qPCR
pSPL3-qPCR-R	GGTCGACTCTAGAGCACGTG	Minigene-derived transcript RT-qPCR
ACTB-F	CATGTACGTTGCTATCCAGGC	Reference gene for RT-qPCR normalization
ACTB-R	CTCCTTAATGTCACGCACGAT	Reference gene for RT-qPCR normalization

Supplementary Table S2. ACMG/AMP evidence framework applied to COL4A3 c.3418+1G>T before and after the functional splicing assay.

Criterion	Initial clinical report	Post-assay assessment	Rationale and limitations
**PVS1**	PVS1_Moderate	PVS1_Moderate (primary); PVS1_Strong (RNA) considered only as a conditional author-level alternative	Minigene assays confirmed complete exon 39 skipping, producing an 81-bp/27-aa in-frame deletion (~1.6% of α3(IV)). The generic size-based pathway supports PVS1_Moderate, which was adopted as primary. PVS1_Strong(RNA) was considered only conditionally because the deleted segment lies within the collagenous domain and contains nine regularly spaced glycine residues; no exon 39-specific expert-panel rule, patient-derived RNA, or direct protein-level evidence was available.
**PM2**	PM2_Supporting	PM2_Supporting retained	Retained from the original clinical report based on absence from the evaluated population databases, including gnomAD.
**PP4**	PP4 (Supporting)	Not applied in the primary classification; PP4 (Supporting) considered only in the conditional alternative	Not applied in the primary classification because isolated glomerular microscopic hematuria has limited specificity and did not co-segregate with the variant in the mother; considered only as Supporting evidence in the conditional alternative.
**Overall classification**	PVS1_Moderate + PM2_Supporting + PP4(Supporting) → Variant of Uncertain Significance	Primary: PVS1_Moderate + PM2_Supporting → VUS<br><br>Conditional alternative: PVS1_Strong (RNA) + PM2_Supporting + PP4 (Supporting) → Likely Pathogenic	Primary conservative classification: VUS. Likely Pathogenic is reached only under the conditional author-level alternative. Neither assessment replaces an accredited clinical laboratory report.

Notes: PVS1(RNA) was used to capture the experimentally observed splicing consequence; PS3 was not applied separately to avoid double counting. PP3 was not independently applied because splice-site disruption was incorporated within the PVS1 framework. PM1 was not independently applied because the collagenous-domain context was considered only for conditional calibration of PVS1(RNA) strength. PP1, PS2, and PM6 were not applied because neither informative co-segregation nor a de novo origin could be established. AlphaFold-based structural visualization and exploratory molecular dynamics findings were not assigned ACMG/AMP evidence codes. Framework references: Richards et al. (2015), Abou Tayoun et al. (2018), Savige et al. (2021), and Walker et al. (2023). Abbreviations: NMD, nonsense-mediated decay; VUS, Variant of Uncertain Significance.

Supplementary Table S3. Time-block RMSD summaries of independently initialized 20-ns WT and MUT trajectories.

Model	Replicate	5–10 ns mean RMSD (nm)	10–15 ns mean RMSD (nm)	15–20 ns mean RMSD (nm)
WT	1	0.616	0.651	0.607
WT	2	0.569	0.578	0.555
WT	3	0.638	0.532	0.572
MUT	1	0.781	0.808	0.836
MUT	2	0.622	0.758	1.048
MUT	3	0.906	0.803	0.729

Mean RMSD values were summarized over prespecified 5–10, 10–15, and 15–20 ns intervals for each individual trajectory to assess short-timescale sampling consistency. Overall RMSD behavior showed broadly similar overall ranges across independent replicates, whereas variation among the prespecified time blocks indicated limited short-timescale sampling consistency. These summaries were not interpreted as evidence of full convergence.
